# Preparation and Degradation of Rhodium and Iridium Diolefin Catalysts for the Acceptorless and Base-Free Dehydrogenation of Secondary Alcohols

**DOI:** 10.1021/acs.organomet.1c00068

**Published:** 2021-03-31

**Authors:** María L. Buil, Alba Collado, Miguel A. Esteruelas, Mar Gómez-Gallego, Susana Izquierdo, Antonio I. Nicasio, Enrique Oñate, Miguel A. Sierra

**Affiliations:** † Departamento de Química Inorgánica, Instituto de Síntesis Química y Catálisis Homogénea (ISQCH), Centro de Innovación en Química Avanzada (ORFEO-CINQA), Universidad de Zaragoza-CSIC, 50009 Zaragoza, Spain; ‡ Departamento de Química Orgánica I, Facultad de CC. Químicas, Centro de Innovación en Química Avanzada (ORFEO-CINQA), Universidad Complutense de Madrid, 28040 Madrid, Spain

## Abstract

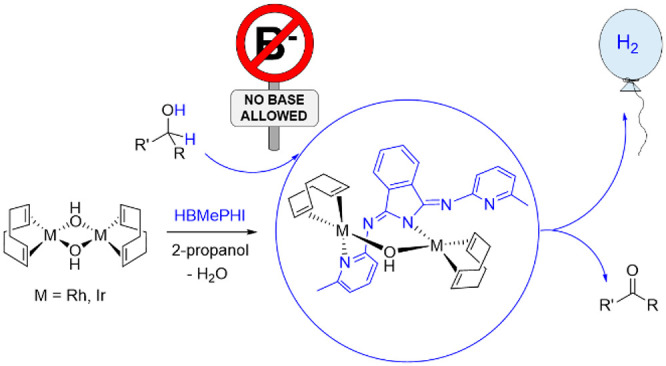

Rhodium and iridium diolefin catalysts for the acceptorless and base-free dehydrogenation of secondary alcohols have been prepared, and their degradation has been investigated, during the study of the reactivity of the dimers [M(μ-Cl)(η^4^-C_8_H_12_)]_2_ (M = Rh (**1**), Ir (**2**)) and [M(μ-OH)(η^4^-C_8_H_12_)]_2_ (M = Rh (**3**), Ir (**4**)) with 1,3-bis(6′-methyl-2′-pyridylimino)isoindoline (HBMePHI). Complex **1** reacts with HBMePHI, in dichloromethane, to afford equilibrium mixtures of **1**, the mononuclear derivative RhCl(η^4^-C_8_H_12_){κ^1^-*N*_py_-(HBMePHI)} (**5**), and the binuclear species [RhCl(η^4^-C_8_H_12_)]_2_{μ-*N*_py_,*N*_py_-(HBMePHI)} (**6**). Under the same conditions, complex **2** affords the iridium counterparts IrCl(η^4^-C_8_H_12_){κ^1^-*N*_py_-(HBMePHI)} (**7**) and [IrCl(η^4^-C_8_H_12_)]_2_{μ-*N*_py_,*N*_py_-(HBMePHI)} (**8**). In contrast to chloride, one of the hydroxide groups of **3** and **4** promotes the deprotonation of HBMePHI to give [M(η^4^-C_8_H_12_)]_2_(μ-OH){μ-*N*_py_,*N*_iso_-(BMePHI)} (M = Rh (**9**), Ir (**10**)), which are efficient precatalysts for the acceptorless and base-free dehydrogenation of secondary alcohols. In the presence of KO^*t*^Bu, the [BMePHI]^−^ ligand undergoes three different degradations: alcoholysis of an exocyclic isoindoline-N double bond, alcoholysis of a pyridyl-N bond, and opening of the five-membered ring of the isoindoline core.

## Introduction

Ketones are a pivotal class of compounds, which can be easily transformed to diverse building blocks including (among others) imines, oximes, amines, and alkenes, the oxidation of alcohols being one of the most representative methods for their preparation.^1^ Traditionally, stoichiometric amounts of chromium- and manganese-based reagents have been used for this purpose.^1a^ As a consequence of the large amounts of noxious waste generated, these methods have been gradually replaced by transition-metal catalysis operating under more environmentally friendly oxidants such as O_2_ and H_2_O_2_.^2^ In the last few years, a further step was taken with the transition-metal-catalyzed acceptorless alcohol dehydrogenation, which does not need the use of oxidants (eq 1). The procedure displays three environmental advantages: it offers an oxidation procedure for the synthesis of carbonyl compounds, minimizing waste formation, it is a promising approach to the production of hydrogen from biomass, and it provides a direct connection with the research on hydrogen storage and transport in organic liquids.^3^ The dehydrogenation of alcohols is generally endothermic at room temperature but can be performed under mild conditions, for instance refluxing toluene in open systems, since the hydrogen elimination acts as a driving force of the reaction.^4^
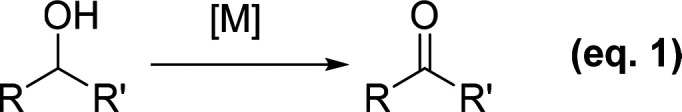
1

Strongly basic media have generally been necessary for the operation of many catalysts, in particular with cationic compounds or precursors bearing halide ligands. The base cocatalyzes the dehydrogenation to generate an alkoxide, which binds to the metal and evolves into the carbonyl compound by β-hydrogen elimination.^5^ To prevent the waste generated by the base, the development of precursors operating under base-free conditions is receiving great attention.^6^ They coordinate ligands, being engaged in the deprotonation step. The basic center usually resides in the first metal coordination sphere^7^ and sometimes in a remote position.^8^

**Scheme 1:**
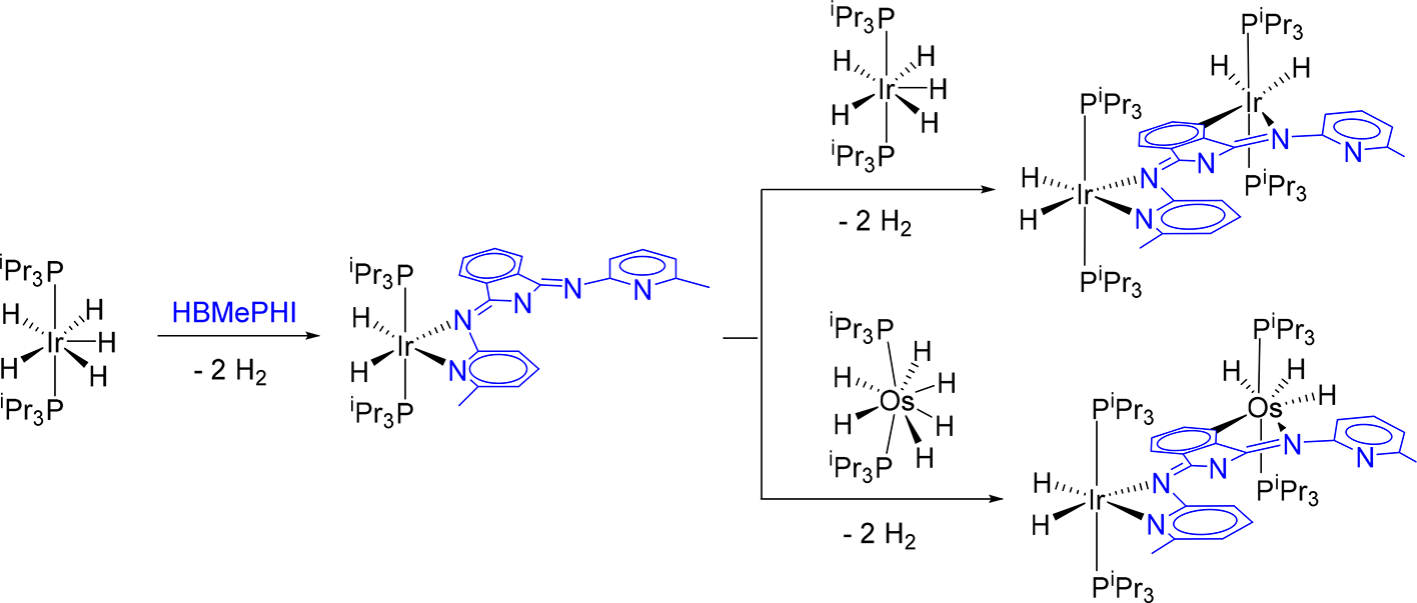
Sequential N–H and C–H Activations of the Isoindoline Core of HBMePHI

We are interested in developing catalysts for the dehydrogenation of hydrogen carriers,^9^ in particular those based on organic liquids.^9f−9h^ Thus, in the search for new precursors, some years ago we initiated a research program based on platinum-group-metal complexes and the polynitrogenated organic molecule 1,3-bis(6′-methyl-2′-pyridylimino)isoindoline (HBMePHI).^10^ Previously, with a few exceptions,^11^ the anion of this isoindoline had been used as a pincer ligand, which modulates the electron density of the metal center and the steric hindrance around it.^12^ However, it is much more than that. We have recently reported that platinum-group-metal polyhydride complexes promote the sequential activations of bonds N–H and C–H of the isoindoline core, to afford homobinuclear and heterobinuclear compounds via mononuclear intermediates (Scheme 1). The bonding of the second metal fragment modifies the electronic structure of the polydentate ligand, which produces a noticeable perturbation of the electron density around the initial center. As a consequence of the mutual electronic influence between the metals, catalytic synergism is observed in the acceptorless and base-free dehydrogenation of secondary alcohols. The bridging ligand displays a noninnocent character, participating in the formation of the metal–alkoxide bond and in the release of molecular hydrogen.^10b^

These unusual findings in the chemistry of pyridylimino-isoindolines prompted us to study the behavior of HBMePHI toward the dimers [M(μ-Cl)(η^4^-C_8_H_12_)]_2_ (M = Rh (**1**), Ir (**2**)) and [M(μ-OH)(η^4^-C_8_H_12_)]_2_ (M = Rh (**3**), Ir (**4**)), which are cornerstones in the development of rhodium^13^ and iridium^14^ organometallic chemistry. This paper reports the results of this study, including the formation of novel eight-membered heterodimetallacycles and C–N bond activations in the isoindoline core, some degradation pathways of the polydentate ligand in basic medium, and the catalytic ability of some of the new complexes in the acceptorless and base-free dehydrogenation of secondary alcohols.

## Results and Discussion

### Reactions with **1** and **2**

**Figure 1:**
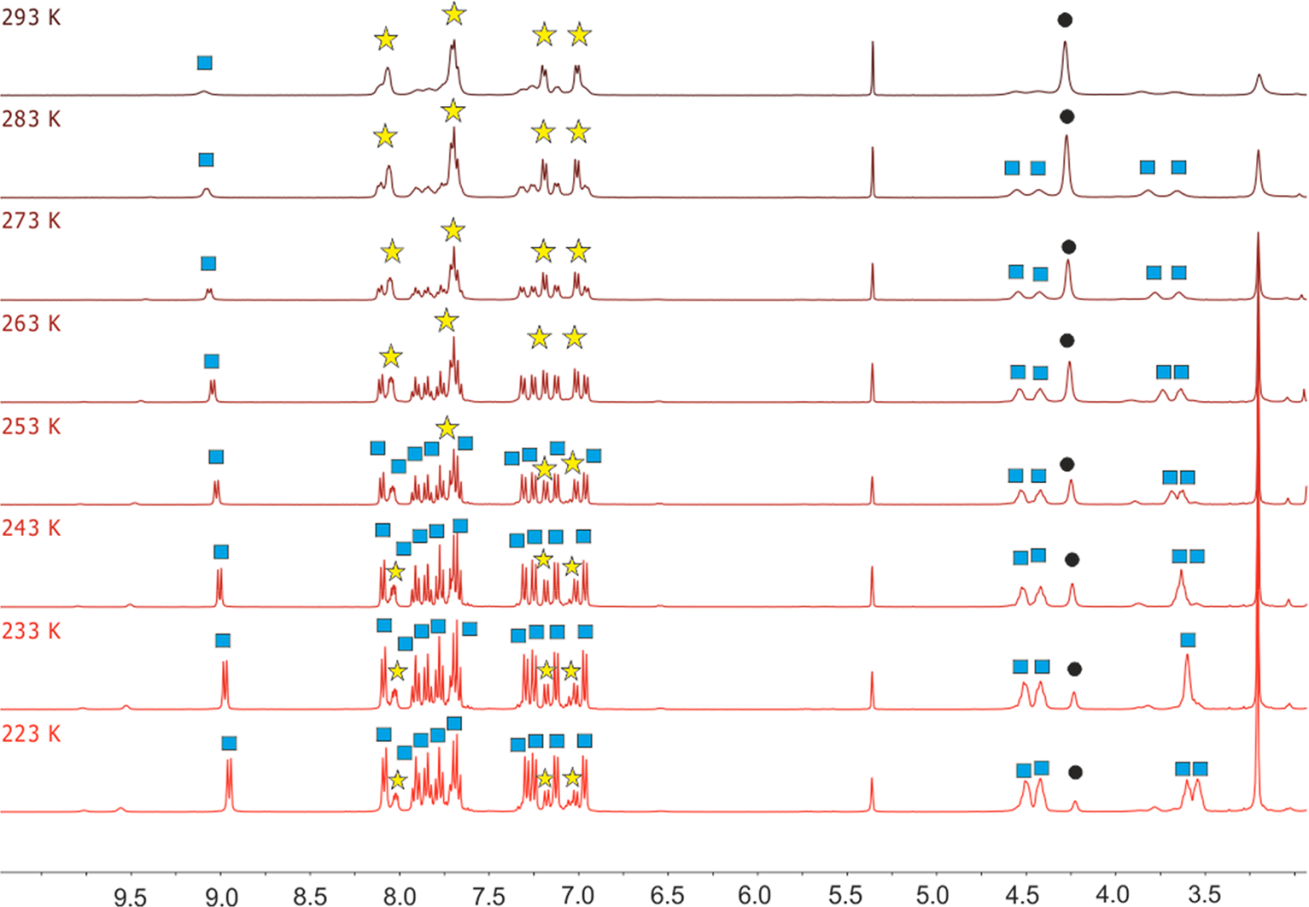
^1^H NMR spectra as a function of the temperature of the equilibrium shown in Scheme 2: blue ■, **5**; yellow ☆, **L**; and black ●, **1** (in CD_2_Cl_2_).

**Scheme 2:**
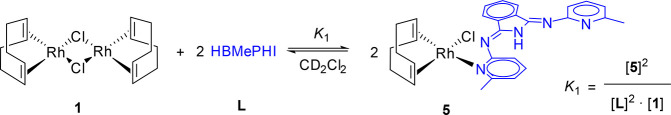
Formation of **5**

**Figure 2:**
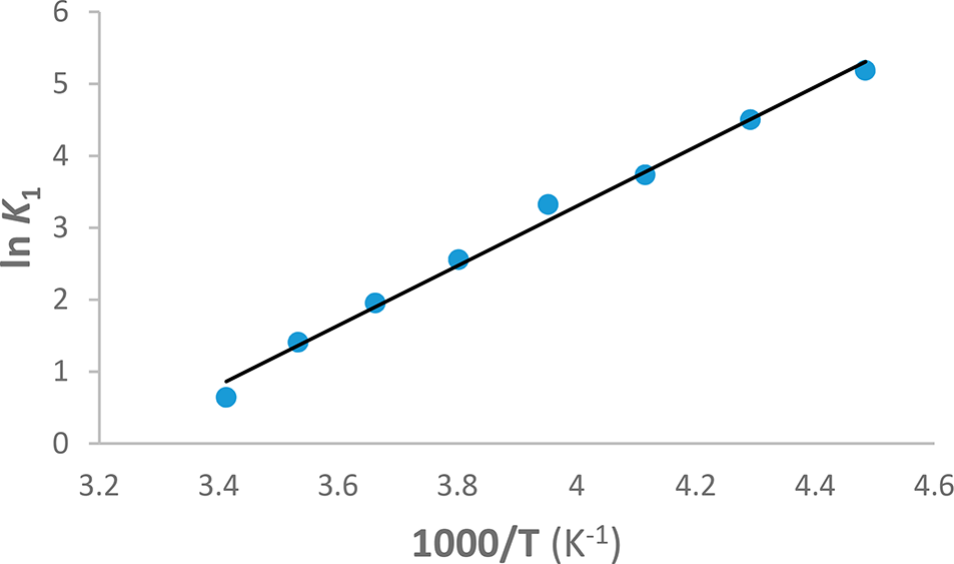
van’t Hoff plot for the equilibrium constant *K*_1_.

The addition of 2.0 mol of HBMePHI to dichloromethane-*d*_2_ solutions of **1** (1.0 equiv per rhodium), contained in an NMR tube, produces a change in the solution color from yellow to orange. The ^1^H NMR spectrum of the mixture at room temperature shows the resonances of **1** and HBMePHI (**L**), which appear slightly broadened, along with markedly broad signals corresponding to a new species. When the sample temperature is lowered, narrowing of all the signals is observed. At the same time, a decrease in the concentrations of both **1** and the isoindoline and an increase in the amount of a new species is clearly evident (Figure 1). Characteristic features of the new compound are 4 resonances between 4.6 and 3.3 ppm due to olefinic hydrogen atoms, which are all inequivalent, and 10 aromatic signals between 9.1 and 6.5 ppm corresponding to the CH hydrogen atoms of the coordinated ligand, which are also inequivalent. In agreement with the ^1^H NMR spectrum, the ^13^C{^1^H} NMR spectrum at 213 K of the new complex displays 4 doublets (^1^*J*_C–Rh_ = 11–13 Hz) between 82 and 74 ppm for the olefinic carbon atoms and 10 aromatic signals for the coordinated isoindoline. These observations can be rationalized according to the equilibrium shown in Scheme 2, which involves the formation of the mononuclear square-planar complex RhCl(η^4^-C_8_H_12_){κ^1^-*N*_py_-(HBMePHI)} (**5**), as a result of the rupture of the chloride bridges of **1** and the coordination of the polydentate molecule to the metal center by one of the pyridyl groups. The equilibrium was studied as a function of the temperature between 293 and 223 K by integration of the olefinic resonances and the higher field aromatic signal of the free ligand. Table 1 collects the values of the equilibrium *K*_1_ constants at each temperature. A linear least-squares analysis of ln *K*_1_ versus 1/*T* (Figure 2) provides values for Δ*H*° and Δ*S*° of −8.2 ± 0.3 kcal mol^–1^ and −26.4 ± 1.0 cal mol^–1^ K^–1^, respectively.

**Table 1:** Formation Constants *K*_1_ and *K*_2_ (L mol^–1^) for **5** and **6**

temp (K)	*K*_1_ (Scheme 2)	*K*_2_ (Scheme 3)
293	1.897	
283	4.077	0.022
273	7.043	0.028
263	12.883	0.04
253	27.692	0.068
243	41.793	0.136
233	89.937	0.242
223	178.606	0.465
213		0.945
203		1.597
193		1.871
183		5.160

**Scheme 3:**
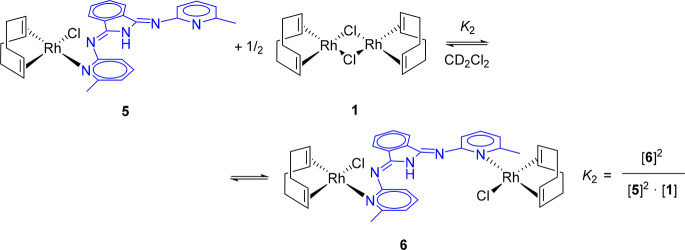
Formation of **6**

**Figure 3:**
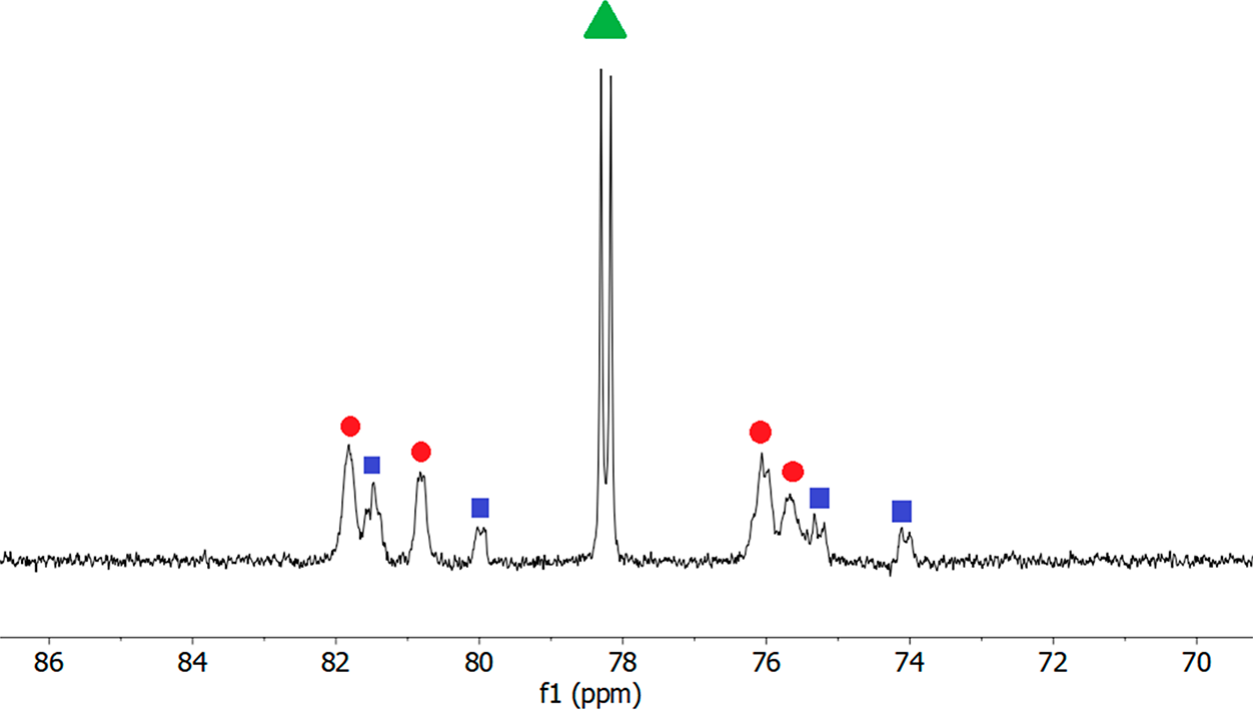
Olefinic resonances in the ^13^C{^1^H} NMR spectrum for the equilibrium shown in Scheme 3: blue ■, **5**; green ▲, **1**; red ●, **6** (183 K, 100.6 MHz, in CD_2_Cl_2_).

**Figure 4:**
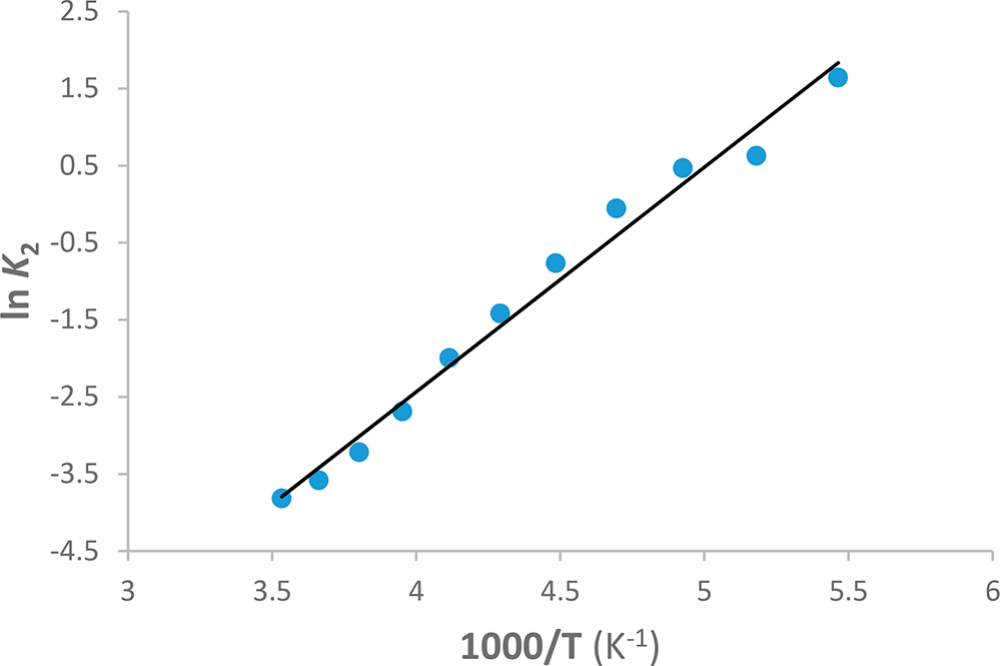
van’t Hoff plot for the equilibrium constant *K*_2_.

The ^1^H and ^13^C{^1^H} NMR spectra of the solutions resulting from the addition of 1.0 mol of HBMePHI per dimer to **1** in dichloromethane-*d*_2_ show significant differences with regard to the previously mentioned spectra. Two noticeable features should be pointed out: the absence of resonances corresponding to the free ligand and the presence of signals due to a new compound. The latter is formed by the reaction of **1** with **5**, and its concentration increases as the sample temperature is decreased. **5** it has four inequivalent olefinic hydrogen atoms. Thus, its ^1^H NMR spectra contain four resonances between 4.6 and 3.4 ppm. Nevertheless, these spectra only show three complex aromatic signals in the 8.2–6.9 ppm range. These observations are consistent with the formation of an equilibrium mixture among **1**, **5**, and the dimer [RhCl(η^4^-C_8_H_12_)]_2_{μ-*N*_py_,*N*_py_-(HBMePHI)} (**6** in Scheme 3). The ^13^C{^1^H} NMR spectra of the mixture are strong additional evidence in favor of this equilibrium. Figure 3 shows the ^13^C{^1^H}-APT spectrum in the olefinic region, at 183 K. The equilibrium shown in Scheme 3 was also studied as a function of the temperature between 283 and 183 K. The thermodynamic parameters obtained from the values of the equilibrium constant *K*_2_ (Table 1) are Δ*H*° = −5.8 ± 0.2 kcal mol^–1^ and Δ*S*°= −28.0 ± 0.7 cal mol^–1^ K^–1^ (Figure 4).

**Scheme 4:**
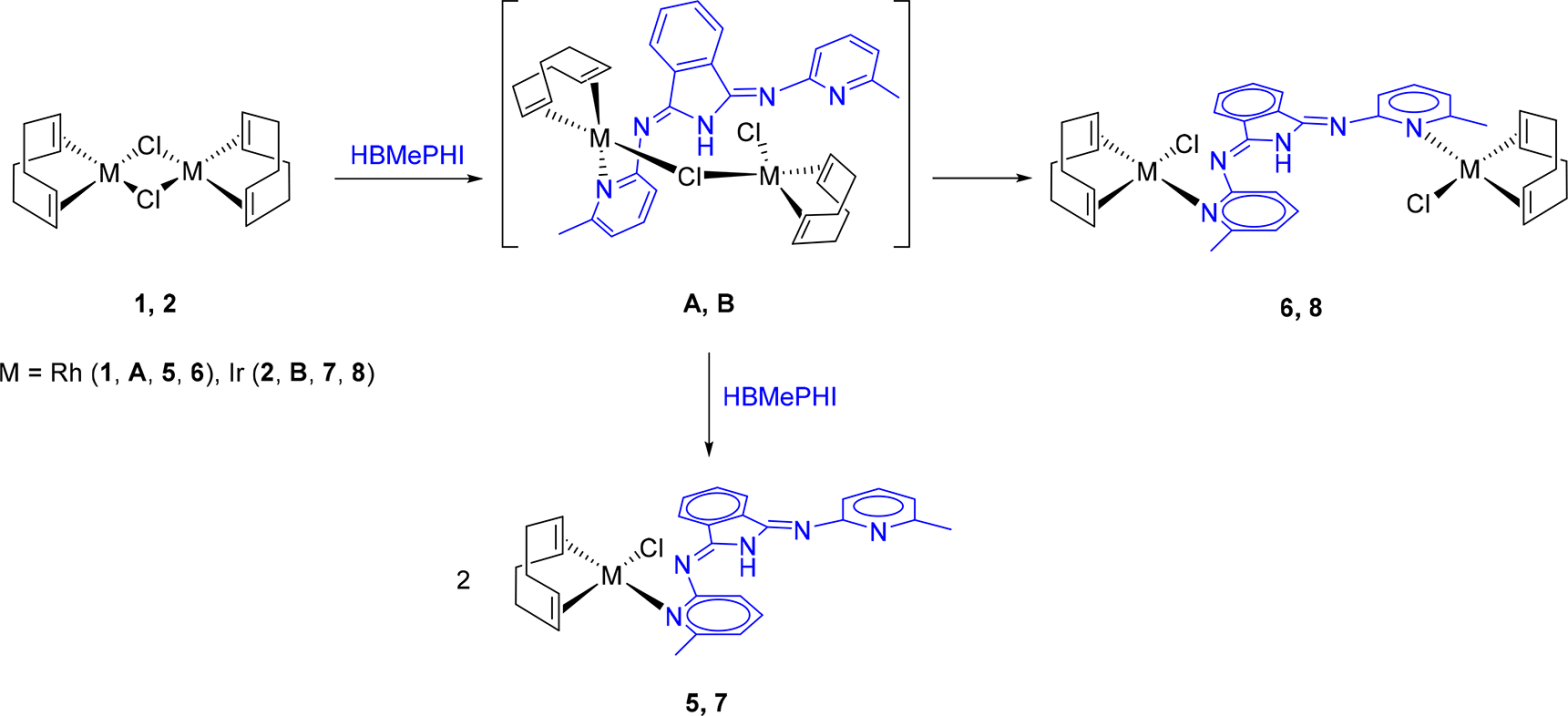
Formation of Complexes **5**–**8**

The iridium dimer **2** also reacts with 1.0 and 0.5 equiv of HBMePHI. The reactions lead to the iridium counterparts of **5** and **6**. These complexes, IrCl(η^4^-C_8_H_12_){κ^1^-*N*_py_-(HBMePHI)} (**7**) and [IrCl(η^4^-C_8_H_12_)]_2_{μ-*N*_py_,*N*_py_-(HBMePHI)} (**8**), are significantly more stable than their rhodium analogues and can be isolated as pure red (**7**) and yellow (**8**) solids in 54% and 80% yields, respectively. The formation of the four compounds might take place via the intermediates (η^4^-C_8_H_12_)ClM(μ-Cl)M{κ^1^-*N*_py_-(HBMePHI)}(η^4^-C_8_H_12_) (M = Rh (**A**), Ir (**B**)), according to Scheme 4.

**Figure 5:**
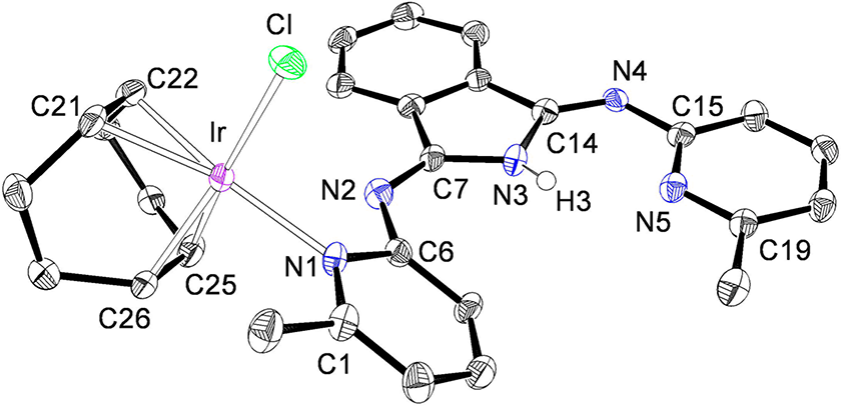
Molecular diagram of complex **7** (50% probability ellipsoids). Hydrogen atoms (except H(3)) are omitted for clarity. Selected bond lengths (Å): Ir–Cl = 2.3608(13), Ir–N(1) = 2.124(4), Ir–C(21) = 2.099(4), Ir–C(22) = 2.112(5), Ir–C(25) = 2.109(5), Ir–C(26) = 2.096(5), N(1)–C(1) = 1.356(6), N(1)–C(6) = 1.368(5), N(2)–C(6) = 1.381(6), N(3)–C(7) = 1.377(6), N(3)–C(14) = 1.400(6), C(21)–C(22) = 1.403(7), C(25)–C(26) = 1.428(7).

**Figure 6:**
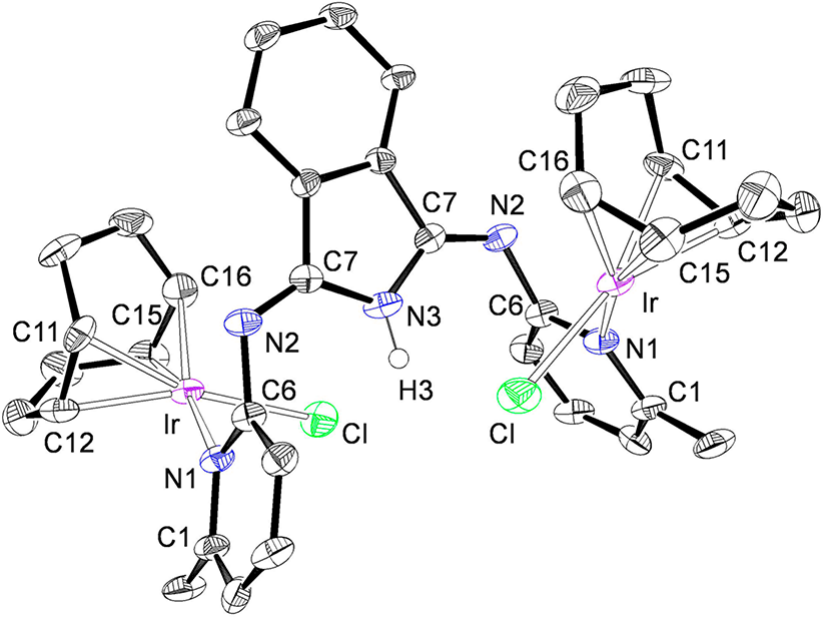
Molecular diagram of complex **8** (50% probability ellipsoids). Hydrogen atoms (except H(3)) are omitted for clarity. Selected bond lengths (Å): Ir–Cl = 2.360(2), Ir–N(1) = 2.111(6), Ir–C(11) = 2.120(7), Ir–C(12) = 2.089(9), Ir–C(15) = 2.137(8), Ir–C(16) = 2.116(8), N(1)–C(6) = 1.338(9), N(2)–C(6) = 1.438(9), N(2)–C(7) = 1.276(9), N(3)–C(7) = 1.386(8), C(11)–C(12) = 1.413(11), C(15)–C(16) = 1.410(12).

Complexes **7** and **8** were characterized by X-ray diffraction analyses. Figure 5 shows the structure of **7**, whereas Figure 6 gives a view of **8**. They confirm the selective coordination of the pyridyl groups of the polydentate HBMePHI molecule and the square-planar environment of the metal centers in these compounds. The coordination gives rise to Ir–N bonds of 2.124(4) Å (Ir–N(1); **7**) and 2.111(6) Å (Ir–N(1); **8**). These bond lengths compare well with those previously reported for other square-planar iridium(I) pyridine derivatives.^15^ The 1,5-cyclooctadiene ligand takes its customary “tub” conformation. The coordinated bonds display distances of 1.403(7) Å (C(21)–C(22)) and 1.428(7) Å (C(25)–C(26)) in **7** and 1.413(11) Å (C(11)–C(12)) and 1.410(12) Å (C(15)–C(16)) in **8**, which are longer than the C–C double bonds in the free diolefin (1.34 Å) in agreement with the usual Chatt–Dewar–Duncanson model.^16^

### Reactions with **3** and **4**

**Scheme 5:**
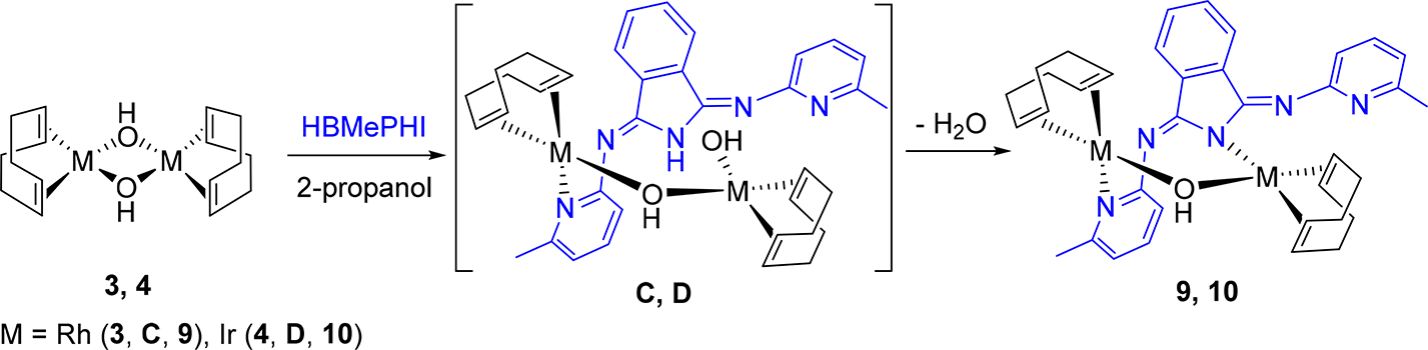
Formation of **9** and **10**

In contrast to the chloride bridging ligand, one of the hydroxide groups of the rhodium dimer **3** is able to abstract the N–H hydrogen atom of HBMePHI. Thus, the treatment of yellow suspensions of this complex, in propan-2-ol, with 1.0 mol of the polydentate molecule for 2 h affords [Rh(η^4^-C_8_H_12_)]_2_(μ-OH){μ-*N*_iso_,*N*_py_-(BMePHI)} (**9**), as a consequence of the asymmetrical coordination of the resulting anion; one pyridyl group coordinates to a rhodium atom, whereas the other metal center is bonded to the N atom of the isoindolinate core. This coordination fashion and the remaining hydroxide group give rise to a mixed double bridge, which generates an eight-membered heterodimetallacycle. Under the same conditions, complex **4** leads to the iridium counterpart [Ir(η^4^-C_8_H_12_)]_2_(μ-OH){μ-*N*_iso_,*N*_py_-(BMePHI)} (**10**). The formation of **9** and **10** should take place via the intermediates (η^4^-C_8_H_12_)(OH)M(μ-OH)M{κ^1^-*N*_py_-(HBMePHI)}(η^4^-C_8_H_12_) (M = Rh (**C**), Ir (**D**)), the hydroxo counterparts of **A** and **B**, according to Scheme 5. Similarly to **1** and **2**, dimers **3** and **4** should initially undergo the rupture of a bridge, by coordination of a pyridyl group of HBMePHI to one of the metal centers. Thus, the subsequent heterolytic N–H activation of the isoindoline core by the other metal center, using the terminal hydroxide group as an internal base, would afford the mixed double bridge. Complexes **9** and **10** were isolated as orange solids in 80% and 47% yields, respectively.

**Figure 7:**
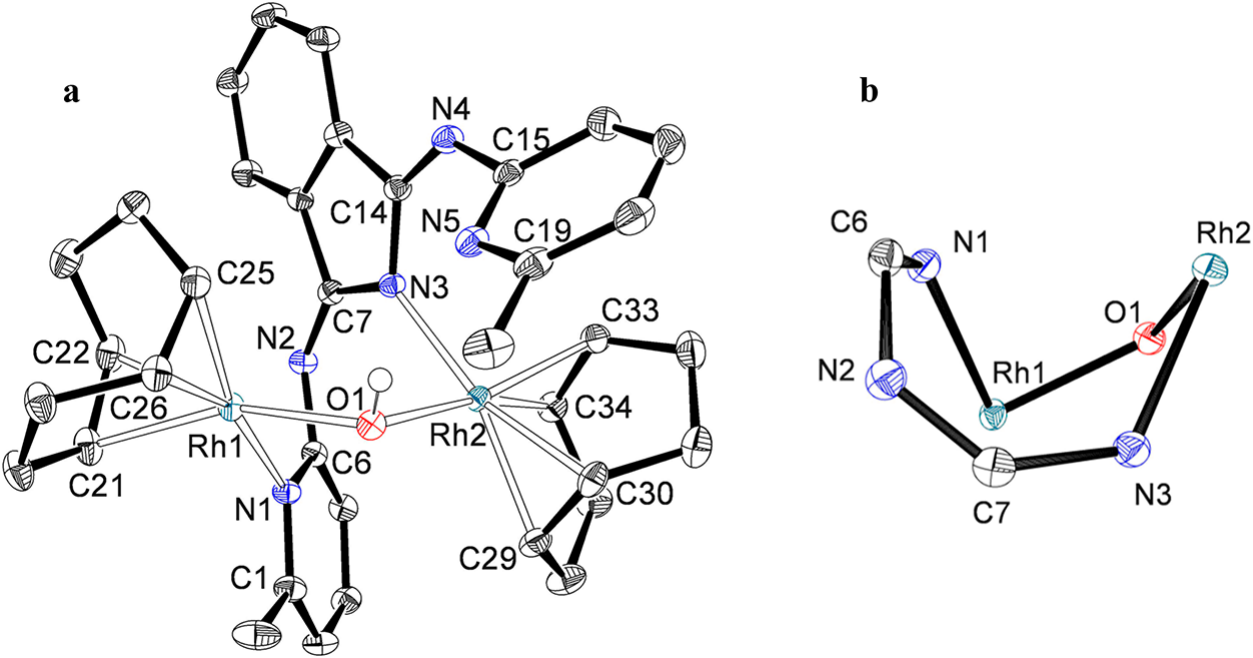
(a) Molecular diagram of complex **9** (50% probability ellipsoids). All hydrogen atoms (except that of the hydroxide ligand) are omitted for clarity. Selected bond lengths (Å) and angles (deg): Rh(1)–O(1) = 2.0709(12), Rh(2)–O(1) = 2.0912(12), Rh(1)–N(1) = 2.1495(14), Rh(2)–N(3) = 2.1047(14), Rh(1)–C(21) = 2.1016(18), Rh(1)–C(22) = 2.1155(18), Rh(1)–C(25) = 2.1075(17), Rh(1)–C(26) = 2.1096(17), N(1)–C(6) = 1.357(2), N(2)–C(6) = 1.382(2), N(2)–C(7) = 1.288(2), N(3)–C(7) = 1.388(2), N(3)–C(14) = 1.391(2), N(4)–C(14) = 1.287(2), N(4)–C(15) = 1.391(2), Rh(1)–O(1)–Rh(2) = 110.63(6), O(1)–Rh(1)–N(1) = 86.48(5), O(1)–Rh(2)–N(3) = 87.63(5). (b) Molecular core.

The rhodium complex **9** was characterized by an X-ray diffraction analysis. The structure (Figure 7) proves the formation of the eight-membered heterodimetallacycle, which displays a boat–boat conformation^17^ with the metals separated by 3.423 Å. The environment around each metal is square-planar, as expected for rhodium(I) centers. The Rh(1)–pyridine distance of 2.1495(14) Å (Rh(1)–N(1)) is about 0.05 Å longer than the Rh(2)–isoindoline bond length of 2.1047(14) Å (Rh(2)–N(3)), suggesting a higher nucleophilicity for the isoindoline N(3) atom than for the pyridine N(1) atom. As a consequence of this, the Rh(1)–hydroxide bond of 2.0709(12) Å (Rh(1)–O(1)) is about 0.02 Å shorter than the Rh(2)–hydroxide bond of 2.0912(12) Å (Rh(2)–O(1)). The lengths of the coordinated C–C double bonds to both metal centers are similar, between 1.393(3) and 1.403(3) Å, and compare well with the distances found in **7** and **8**. Several conformations of similar energy are possible for an eight-membered cycle. As a consequence, complexes **9** and **10** are fluxional in toluene-*d*_8_ solution, showing a rigid structure at temperatures lower than 213 K. In agreement with Figure 7, their ^1^H NMR spectra display eight olefinic resonances between 5.5 and 3.0 ppm, whereas the ^13^C{^1^H} NMR spectra contain eight olefinic signals in the 85–52 ppm range.

The chelate κ^2^-(*N*_iso_,*N*_py_) coordination is known for 1,3-bis(2′-pyridylimino)isoindolate (BPHI) anions.^11^ However, as far as we know, the bridge μ-(*N*_iso_,*N*_py_) coordination is unprecedented. Compounds bearing bridging [BPHI]^−^ ligands are very scarce. Baird and co-workers have observed that HBPHI displaces an acetate group from Mo_2_(OAc)_4_ to give Mo_2_(OAc)_3_(BPHI), with the [BPHI]^−^ ligand bound to one molybdenum by an imino nitrogen and to the other molybdenum by the isoindoline nitrogen and a pyridyl nitrogen.^18^ Bröring and co-workers have reported that one of the pyridyl groups of HBMePHI undergoes a palladium-promoted 1,3-hydrogen shift, from C to N, to afford Pd(κ^3^-*N*_py_,*N*_iso_,*C*_Hpy_)-pincer derivatives, which add a second palladium to the free pyridyl-imine moiety.^19^ We have described the preparation of homoleptic and heteroleptic bis(osmium) complexes containing a [μ-(κ^2^-*N*_py_,*N*_imine_)_2_-BMePHI]^−^ ligand,^10a^ whereas Li, Yang, Zhang, and co-workers have observed the same coordination fashion in an intermediate species formed in the reaction of Lu(CH_2_SiMe_3_)_3_(thf)_2_ with HBPHI to give Lu{κ^3^-*mer*-(BPHI)}(CH_2_SiMe_3_)_2_.^20^

### Degradation of the [BMePHI]^−^ ligand in Basic Medium

Alcohol dehydrogenation catalysts combined with bases promote borrowing-hydrogen reactions, including α-alkylation of arylacetonitriles and methyl ketones.^21^ The carbonyl compound resulting from the dehydrogenation process undergoes a base-catalyzed condensation with an alkyl substrate to afford an α,β-unsaturated intermediate,^22^ which is subsequently reduced to the final product with the hydrogen generated in the dehydrogenation.^21^ In order to explore the ability of the Rh- and Ir(BMePHI)(diolefin) systems to work in this class of catalysis, we studied the formation of **9** and **10** in the presence of a strong base.

**Scheme 6:**
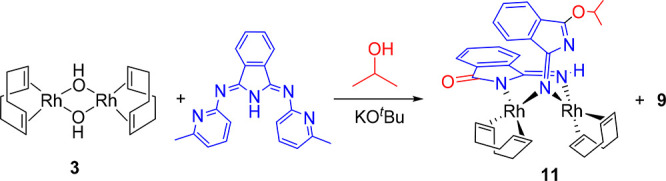
Formation of **11**

Treatment of a suspension of **3** in propan-2-ol with 2.0 mol of HBMePHI and 3.0 equiv of KO^t^Bu at room temperature for 2 h leads to a mixture of **9** and [Rh(η^4^-C_8_H_12_)]_2_{μ-*N*_iso_,*N*_imine_-(HN=C_8_H_4_NO)}(μ-N=C_8_H_4_NO^*i*^Pr) (**11**), according to Scheme 6.

**Figure 8:**
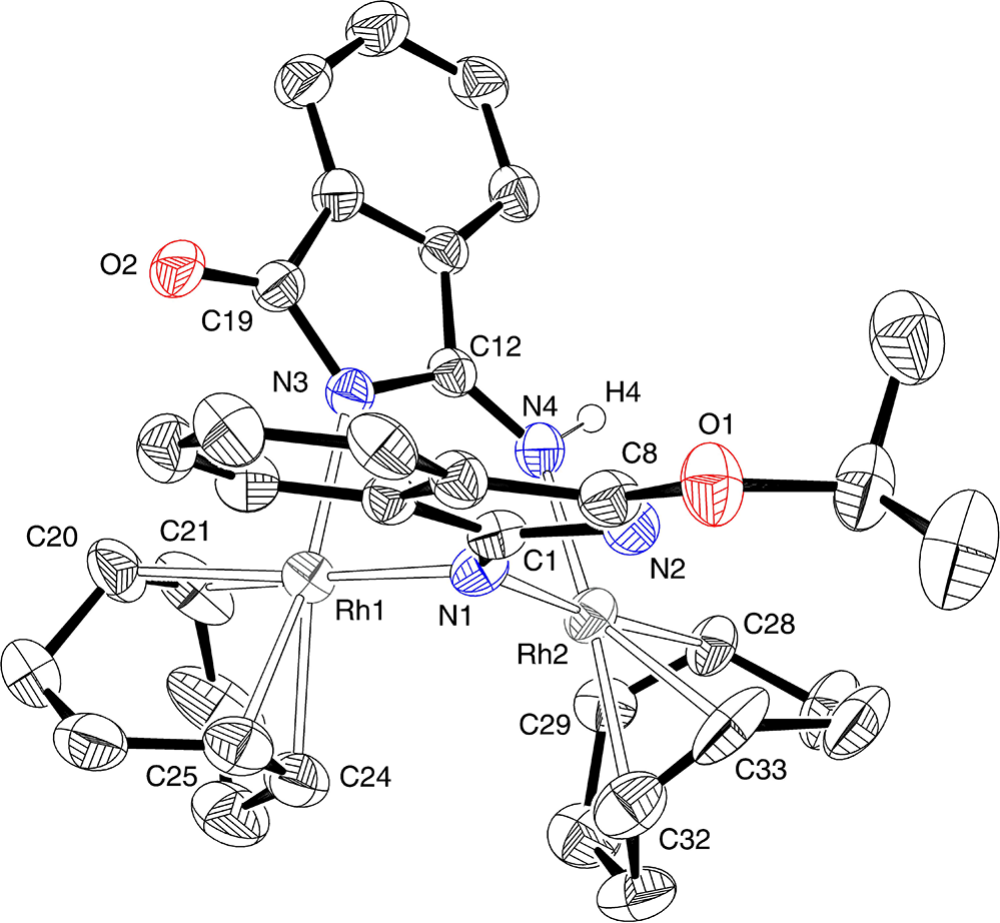
Molecular diagram of complex **11** (50% probability ellipsoids). All hydrogen atoms (except the imine hydrogen atom H4) are omitted for clarity. Selected bond lengths (Å) and angles (deg): Rh(1)–N(1) = 2.048(3), Rh(1)–N(3) = 2.090(3), Rh(2)–N(1) = 2.049(3), Rh(2)–N(4) = 2.106(4), Rh(1)–C(20) = 2.134(5), Rh(1)–C(21) = 2.125(5), Rh(1)–C(24) = 2.147(5), Rh(1)–C(25) = 2.125(5), Rh(2)–C(28) = 2.155(4), Rh(2)–C(29) = 2.115(5), Rh(2)–C(32) = 2.160(5), Rh(2)–C(33) = 2.106(5), N(1)–C(1) = 1.260(5), N(2)–C(1) = 1.449(5), N(2)–C(8) = 1.286(5), N(3)–C(12) = 1.364(5), N(3)–C(19) = 1.371(5), N(4)–C(12) = 1.292(5), O(1)–C(8) = 1.334(5), O(1)–C(9) = 1.471(5), O(2)–C(19) = 1.236(5). Rh(1)–N(1)–Rh(2) = 96.93(14).

Complexes **9** and **11** were separated by using their different solubilities in propan-2-ol. Thus, complex **11** was obtained pure in 20% yield with regard to **3** as red crystals suitable for X-ray diffraction analysis. Its structure (Figure 8) reveals the formation of a surprising mixed double bridge. One of the halves of the bridge is the anion resulting from the deprotonation of the NH-isoindoline group of 3-iminoisoindolin-1-one (HN=C_8_H_4_NHO), which coordinates different metal centers in an *N*_iso_,*N*_imine_ fashion, whereas the other half is the azavinylidene resulting from the deprotonation of the NH-imine unit of 3-isopropoxy-1*H*-isoindol-1-imine (HN=C_8_H_4_NO^i^Pr). The formation of the [HN=C_8_H_4_NO]^−^ anion involves two different alcoholysis processes in the imine moieties of a [BMePHI]^−^ ligand. The C=O double bond could be the result of the substitution of a pyridylimine moiety by two isopropoxide groups. Then, the resulting diisopropylacetal intermediate^23^ should lose diisopropyl ether to afford the carbonyl group.^24^ In contrast, the other imino group undergoes alcoholysis of the imine–pyridyl bond. The azavinylidene bridge ([N=C_8_H_4_NO^i^Pr]^−^) arises from a similar process involving two alcoholyses on the imine functions of a second [BMePHI]^−^ ligand. The main difference between the generation processes of both bridges is the number of molecules of propan-2-ol attacking the imine–isoindoline bond. When only a molecule of propan-2-ol attacks, the isopropoxide group remains as a substituent at the 2-position of the isoindoline core. Its steric hindrance prevents the coordination of the isoindoline-N atom, whereas the electronic difference with the carbonyl oxygen atom seems to favor the deprotonation of the imine resulting from the alcoholysis of the imine–pyridyl bond.

The rhodium atoms display square-planar coordination environments with a separation between the metals of 3.0672(5) Å, which is long for a single Rh–Rh bond. In agreement with this, overlap between their d_*z*^2^_ orbitals has not been found by means of DFT calculations (M06/6-311G(d,p)&SDD(f)). Although the isoindoline coordination of the anion [HN=C_8_H_4_NO]^−^ to Rh(1) of 2.090(3) Å (Rh(1)–N(3)) is about 0.01 Å shorter than the imine coordination to Rh(2) of 2.106(4) Å (Rh(2)–N(4)), the azavinylidene–rhodium bond lengths of 2.048(3) Å (Rh(1)–N(1)) and 2.049(3) Å (Rh(2)–N(1)) are statistically identical and similar to those reported for the complex [Rh(μ-N=CPh_2_)(TFB)]_2_ (TFB = tetrafluorobenzobarrelene; 2.046(7), 2.052(7), 2.054(6) and 2.054(7) Å).^25^ The M–azavinylidene–M angle, Rh(1)–N(1)–Rh(2), of 96.93(14)° and the distance N(1)–C(1) of 1.260(5) Å compare well with those found in other transition-metal compounds bearing azavinylidene bridges.^26^ The lengths of the coordinated C=C double bonds, between 1.353(8) and 1.387(7) Å, are slightly shorter than those found in **7** and **8**. The ^1^H and ^13^C{^1^H} NMR spectra of **11** in benzene-*d*_6_ at room temperature are consistent with the structure shown in Figure 8. The ^1^H NMR spectrum displays a broad signal at 5.78 ppm corresponding to the imine-NH hydrogen atom and eight olefinic resonances due to the inequivalent C_sp^2^_-H hydrogen atoms of the diene, between 6.5 and 3.3 ppm, whereas the ^13^C{^1^H} NMR spectrum contains eight doublets (^1^*J*_C–Rh_ = 10–13 Hz) between 87 and 74 ppm, assigned to the coordinated carbon atoms.

**Figure 9:**
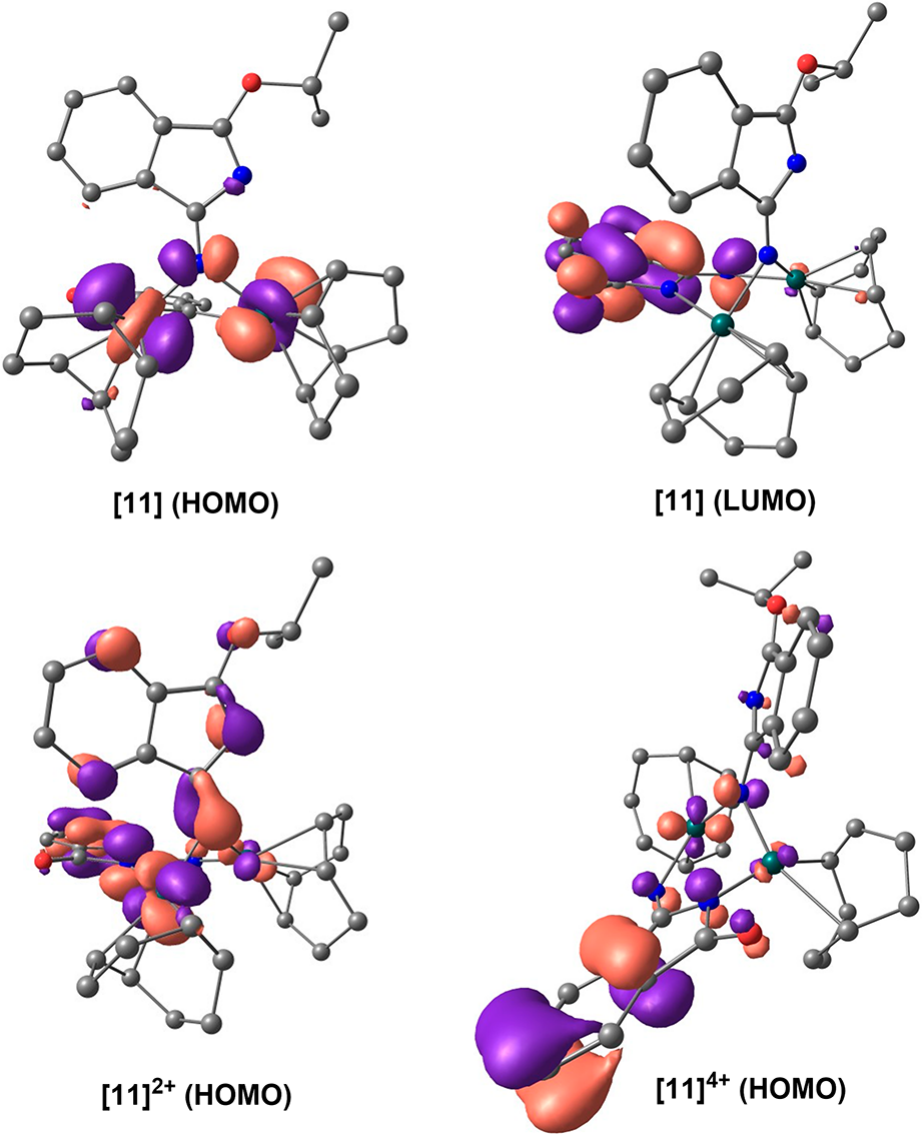
Computed (DFT/M06/6-311G(d,p)&SDD(f)) HOMO and LUMO orbitals for complex **11** and for the oxidation products dication [**11**]^2+^ and tetracation [**11**]^4+^. Hydrogen atoms are omitted for clarity. The isosurface value is 0.043.

The study of the electrochemistry of binuclear complexes is always attractive due to the possible interaction between the two metals. Unfortunately, complexes **9** and **10** were unstable and decomposed in the electrode, but the cyclic voltammetry of **11** was conducted under an argon atmosphere in dry, oxygen-free dichloromethane (10^–3^ M analyte concentration) containing [N^n^Bu_4_]PF_6_ as the supporting electrolyte (10^–1^ M) and using a Ag/AgCl reference electrode (3 M, KCl). Under these conditions complex **11** displays two oxidation events, one of them quasi-reversible at 0.49 V and the second one irreversible at 1.10 V (Table 2 and Figures S1–S3). The DFT (M06/6-311G(d,p)&SDD(f)) calculations reveal that the HOMO of the complex is equally distributed between the metal centers, whereas the LUMO is located in the isoindolinate [HN=C_8_H_4_NO]^−^ ligand. The loss of one electron by each metal (two electrons per molecule) leads to the dication [**11**]^2+^, which also has the HOMO mainly centered on the metals, although some participation of the coordinated isoindolinate anion is observed. The subsequent loss of two electrons affords the tetracation [**11**]^4+^, having the HOMO mainly centered on the isoindolinate ligand (Figure 9). These data suggest that the oxidation events are compatible with two sequential processes of two electrons: from Rh^I^L_2_Rh^I^ to Rh^II^L_2_Rh^II^ and from Rh^II^L_2_Rh^II^ to Rh^III^L_2_Rh^III^. The successive oxidations give rise to the approach of the metal centers to 2.780 Å in the dication and to 2.747 Å in the tetracation (Figure S6). Although these distances lie within the range of distances assumed for a Rh–Rh single bond (2.62–2.84 Å),^27^ overlapping between the d orbitals of the metals is not observed.

**Table 2:** Oxidation Potentials of Complexes **11** and **12**^a^

complex	*E*_pa1_	*E*_pa2_	*E*_pa3_
**11**	0.49^b^ (*E*_pc1_ 0.39) (Δ*E* 95 mV)	1.10	
**12**	0.56	0.74	1.37

a Data obtained from dichloromethane solutions of **11** and **12** (10^−3^M), containing (NBu_4_)PF_6_ (10^–1^ M) as the supporting electrolyte at 20 °C: counter electrode, Pt; working electrode, glassy carbon; reference electrode, Ag/AgCl; scan rate, 100 mV/s. Values are given in V and referenced vs Ag/AgCl.

b Quasi-reversible wave.

**Scheme 7:**
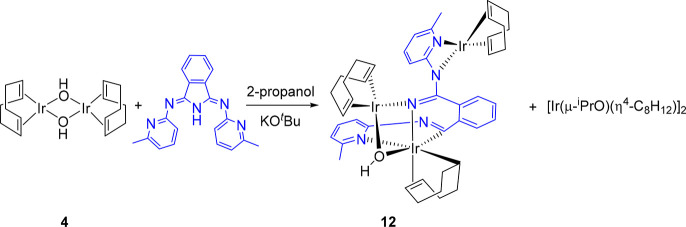
Formation of **12**

The Ir(BMePHI)(diolefin) systems are also unstable in basic medium. As in the rhodium case, the instability is associated with the reactivity of the [BMePHI]^−^ ligand in basic medium, which is strongly directed by the metal center. Under the same conditions as those giving rise to the mixture of **9** and **11**, dimer **4** affords a mixture of the isopropoxide dimer [Ir(μ-O^*i*^Pr)(η^4^-C_8_H_12_)]_2_ and the trinuclear derivative Ir_3_(η^4^-C_8_H_12_)_2_(κ^1^-*C*,η^2^-C_8_H_13_)(μ-OH)(L) (**12** in Scheme 7). Using complex **10** as a reference, the L ligand of **12** can be described as the result of the oxidative addition of the C–N bond, substituted with the free pyridyl-imine group, of the five-membered ring of the isoindoline core to the pyridyl-coordinated metal center. The addition of a hydride to one of the C–C double bonds of the diene coordinated to the generated iridium(III) center and the addition of an [Ir(η^4^-C_8_H_12_)]^+^ fragment to the free pyridyl-imine group give rise to this novel molecule. The metal-promoted degradation of the five-membered heterocycle of an isoindoline is certainly notable. In this context, it should be highlighted that the isoindoline skeleton is a part of a large variety of biologically active synthetic compounds, which have a wide range of applications in medicine.^28^

**Figure 10:**
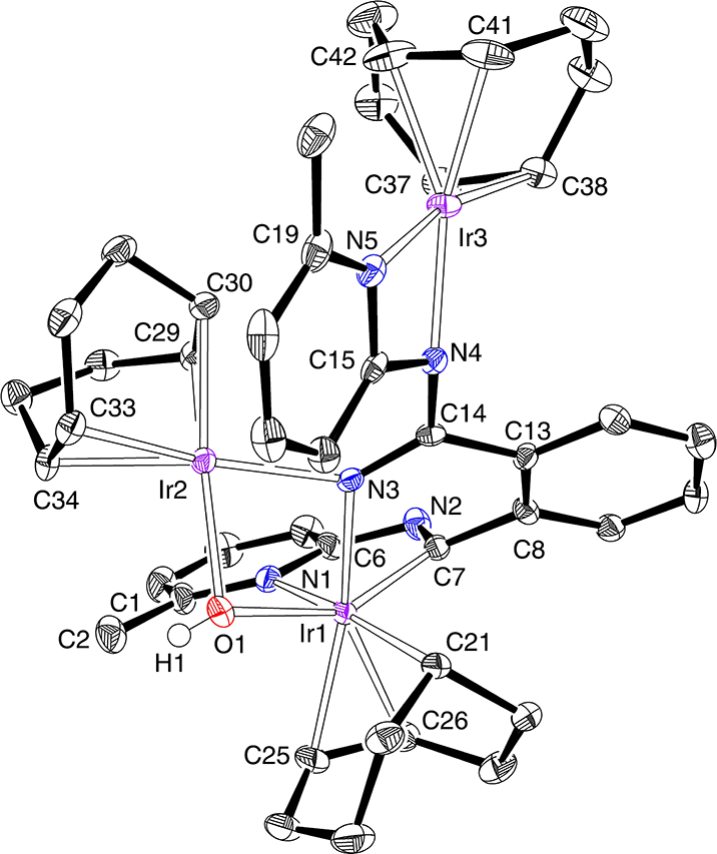
Molecular diagram of complex **12** (50% probability ellipsoids). All hydrogen atoms (except that of the hydroxide ligand) are omitted for clarity. Selected bond lengths (Å) and angles (deg): Ir(1)–C(7) = 1.982(4), Ir(1)–N(3) = 2.047(3), Ir(1)–N(1) = 2.236(4), Ir(1)–O(1) = 2.253(3), Ir(1)–C(21) = 2.102(5), Ir(1)–C(25) = 2.163(4), Ir(1)–C(26) = 2.172(5), Ir(2)–N(3) = 2.035(3), Ir(2)–O(1) = 2.072(3), Ir(2)–C(29) = 2.071(4), Ir(2)–C(30) = 2.120(4), Ir(2)–C(33) = 2.101(4), Ir(2)–C(34) = 2.130(4), Ir(3)–N(4) = 2.072(3), Ir(3)–C(37) = 2.096(4), Ir(3)–C(38) = 2.111(5), Ir(3)–C(41) = 2.114(5), Ir(3)–C(42) = 2.092(5), N(1)–C(1) = 1.367(5), N(1)–C(6) = 1.370(5), N(2)–C(6) = 1.396(5), N(2)–C(7) = 1.301(5), N(3)–C(14) = 1.296(5), N(4)–C(14) = 1.403(5), N(4)–C(15) = 1.379(5), N(5)–C(15) = 1.357(5), N(5)–C(19) = 1.357(5), C(21)–Ir(1)–N(1) = 169.13(15), N(1)–Ir(1)–C(7) = 74.85(15), C(7)–Ir(1)–O(1) = 151.58(14), O(1)–Ir(1)–N(3) = 73.32(12), N(4)–Ir(3)–N(5) = 63.61(13).

**Chart 1:**
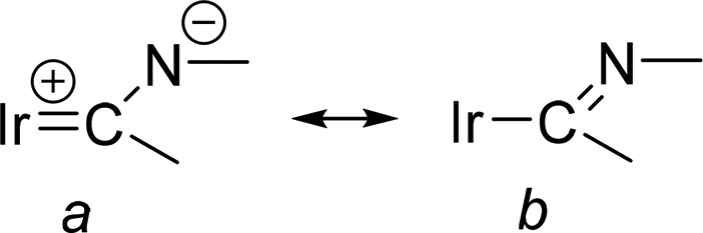
Resonance for the Ir(1)–C(7) Bond

Complex **12** was separated from the mixture by extraction in toluene and crystallized pure in 22% yield with regard to **4** as orange crystals suitable for X-ray diffraction analysis. Its structure (Figure 10) proves the degradation of the [BMePHI]^−^ ligand and the trinuclear nature of the complex, which is formed by an octahedral iridium(III) center (Ir(1)) and two square-planar iridium(I) centers (Ir(2) and Ir(3)). The octahedron around Ir(1) is defined by two chelates and a hydroxide-azavinylidene double bridge. The chelate C_8_H_13_-carbocycle coordinates with three different Ir–C distances, as expected for the κ^1^-*C*,η^2^-coordination, which compare well with those reported for other complexes bearing C_8_H_12_R rings similarly linked to iridium(III).^29^ The σ-Ir(1)–C(21) single bond of 2.102(5) Å is about 0.06 and 0.07 Å shorter than the metal–olefin bonds Ir(1)–C(25) and Ir(1)–C(26) of 2.163(4) and 2.172(5) Å, respectively. The Ir(1)–C(21) bond is disposed *trans* to the pyridyl group of a (C(7),N(1))-iminyl-pyridine moiety (C(21)–Ir(1)–N(1) = 169.13(15)°), which has a N(1)–Ir(1)–C(7) bite angle of 74.85(15)°. The iridium–pyridine distance of 2.236(4) Å (Ir(1)–N(1)) is slightly longer than those found in **7** and **8**, whereas the Ir(1)–C(7) bond length of 1.982(4) Å is about 0.02 Å shorter than the Ir(1)–C(21) single bond and even shorter than those reported for other iridium-iminyl derivatives.^30^ This suggests that, for an adequate description of the Ir(1)–C(7) bonding situation, the resonance form *a* shown in Chart 1 should also be taken into account. Atom C(7) is disposed *trans* to the hydroxide group with a C(7)–Ir(1)–O(1) angle of 151.58(14)°, while the other part of the double bridge, the azavinylidene ligand, lies *trans* to the C(25)–C(26) double bond. The double bridge and the Ir(2) atom form a metalloligand, which coordinates to Ir(1) with an O(1)–Ir(1)–N(3) bite angle of 73.32(12)°. The iridium–azavinylidene distances of 2.047(3) Å (Ir(1)–N(3)) and 2.035(3) Å (Ir(2)–N(3)) are statistically identical. However, the Ir(1)–O(1) distance of 2.253(3) Å is about 0.17 Å longer than the Ir(2)–O(1) bond length of 2.072(3) Å. The *N*_imine_*,N*_py_ chelate coordinates to Ir(3) with a N(4)−Ir(3)−N(5) bite angle of 63.61(13)°, which compares well with the reported angles for the κ^2^-*N*_py_,*N*_imine_-coordination of the [BMePHI]^−^ anion.^10^ The coordinated C=C double bonds display distances between 1.299(6) and 1.429(6) Å. The asymmetry of **12** is also evident in the ^1^H and ^13^C{^1^H} NMR spectra. Thus, the former shows 10 olefinic resonances between 5.6 and 3.0 ppm, due to the inequivalent C_*sp2*_H-hydrogen atoms of the carbocycles, in addition to the signals corresponding to the Ir(1)C(21)H- and OH-hydrogen atoms, which appear at 0.65 and 0.24 ppm, respectively. The ^13^C{^1^H} NMR spectrum agrees with the ^1^H NMR spectrum. Thus, it contains 10 olefinic resonances between 86.5 and 32.3 ppm. The signal corresponding to the carbocyclic C(21) atom is observed at 32.1 ppm, whereas that due to the iminyl C(7) atom appears at 198.9 ppm. This chemical shift, at an unusually low field, is more evidence for a significant contribution of the resonance form *a* to the Ir(1)–C(7) bond.

The electrochemical behavior of binuclear complex **12** is summarized in Table 2. As shown in Figures S4 and S5, it undergoes three irreversible oxidations at 0.56, 0.74, and 1.37 V. The DFT (M06/6-311G(d,p)&SDD(f)) calculations reveal that the HOMO of the molecule is mainly located on the iridium(I) center Ir(2), whereas the LUMO is distributed along the octahedral iridium(III) center Ir(1) and its associated ligands (Figures S7–S9). After the loss of one electron, the spin density of the molecule is still localized on Ir(2) (computed spin density 0.71 e^–^). Thus, it is reasonable to think that the second oxidation also takes place on this center. Thus, the peaks at 0.56 and 0.74 V can be assigned to the sequential oxidations of Ir(2), from Ir(I) to Ir(II) and from Ir(II) to Ir(III). It is likely that the third oxidation at 1.37 V could correspond to the other iridium(I) center, Ir(3). Overall, the oxidation of **12** is mainly determined by the oxidation states of the metal centers, which behave independently from each other.

### Aceptorless and Base-Free Dehydrogenation of Secondary Alcohols Catalyzed by **9** and **10**

The reactions were performed in toluene at 100 °C, using a substrate concentration of 0.255 M and a catalyst concentration of 9.0 × 10^–3^ M (i.e., 7 mol % of the metal). Table 3 collects the alcohols studied and the yield of carbonyl compounds formed as a function of the catalyst after 24 h.

**Table 3:**
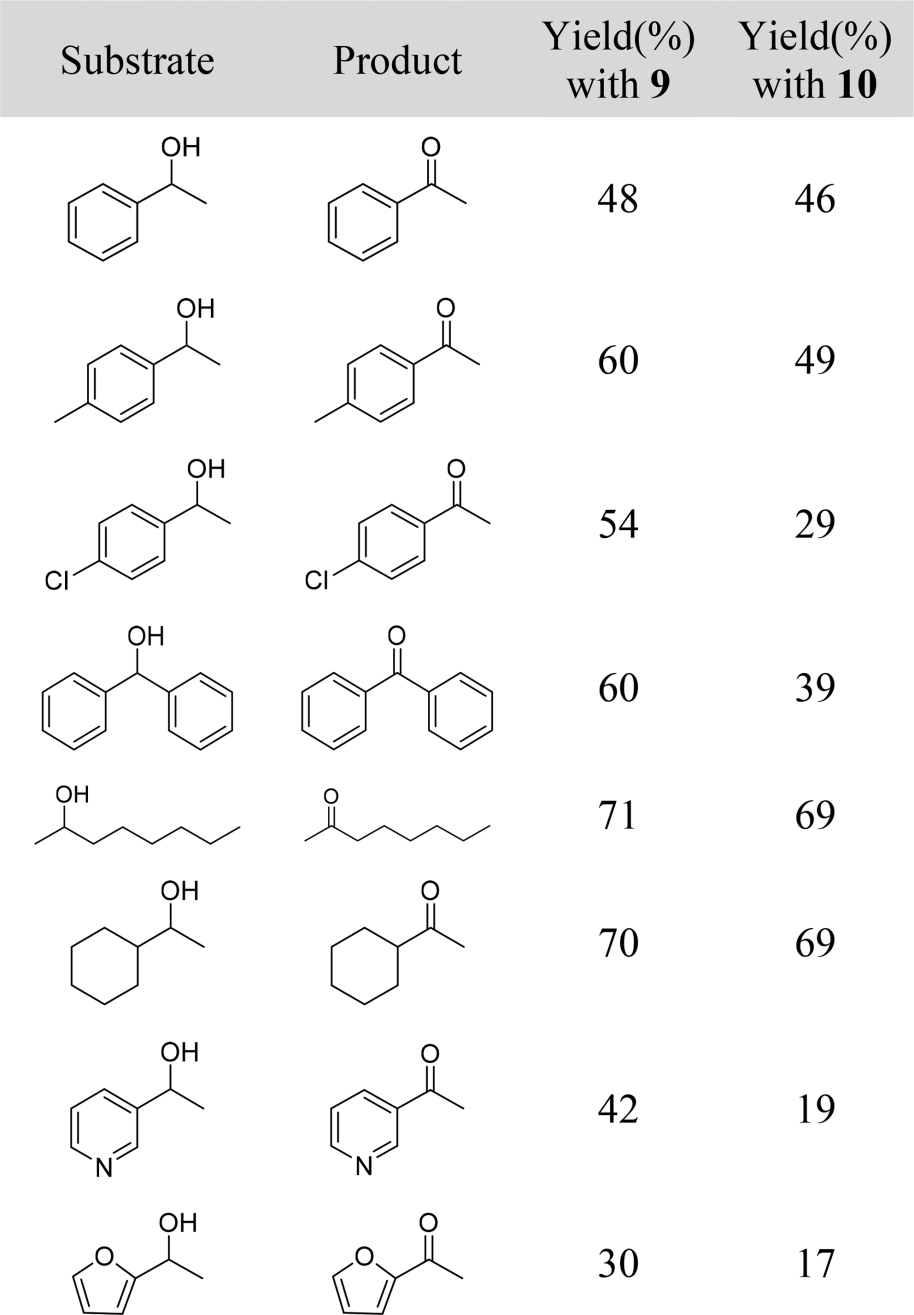
Metal-Mediated Acceptorless and Base-Free Dehydrogenation of a Secondary Alcohol^a^

a Conditions: complex **9** or **10** (0.009 mmol); substrate (0.255 mmol); toluene (1 mL); heated at 100 °C for 24 h. Conversions were calculated from the relative peak area integrations of the reactant and product in the GC spectra.

Ketones are obtained in moderate to high yields after 24 h. Both catalysts are more efficient for the dehydrogenation of aliphatic alcohols in comparison to that for benzylic or benzhydrylic alcohols. Thus, while ketones resulting from the dehydrogenation of substrates such as 1-phenylethanol, 3-pyridylethanol, 1-(2-furyl)ethanol, and diphenylmethanol are obtained in 20–60% yield, 2-octanol and 1-cyclohexylethanol are dehydrogenated in about 70% yield. Complexes **9** and **10** are even more efficient than the binuclear polyhydrides shown in Scheme 1, (P^*i*^Pr_3_)_2_H_2_Ir{μ-(κ^2^-*N*_py_,*N*_imine_-BMePI-κ^2^-*N*_imine_,*C*^4^_iso_)}IrH_2_(P^*i*^Pr_3_)_2_ and (P^*i*^Pr_3_)_2_H_2_Ir{μ-(κ^2^-*N*_py_,*N*_imine_-BMePI-κ^2^-*N*_imine_,*C*^4^_iso_)}OsH_3_(P^*i*^Pr_3_)_2_, for the dehydrogenation of aliphatic alcohols.^10b^ This ability is in contrast to the generally observed trend. Aromatic groups stabilize the ketone and appear to increase the dehydrogenation rate of the alcohol. The rhodium complex **9** is significantly more efficient than the iridium derivative **10** for the dehydrogenation of aromatic substrates, in particular for 3-pyridylethanol, 1-(2-furyl)ethanol, and diphenylmethanol, whereas the oxidation of aliphatic alcohols occurs with similar efficiency in the presence of both complexes.

**Scheme 8:**
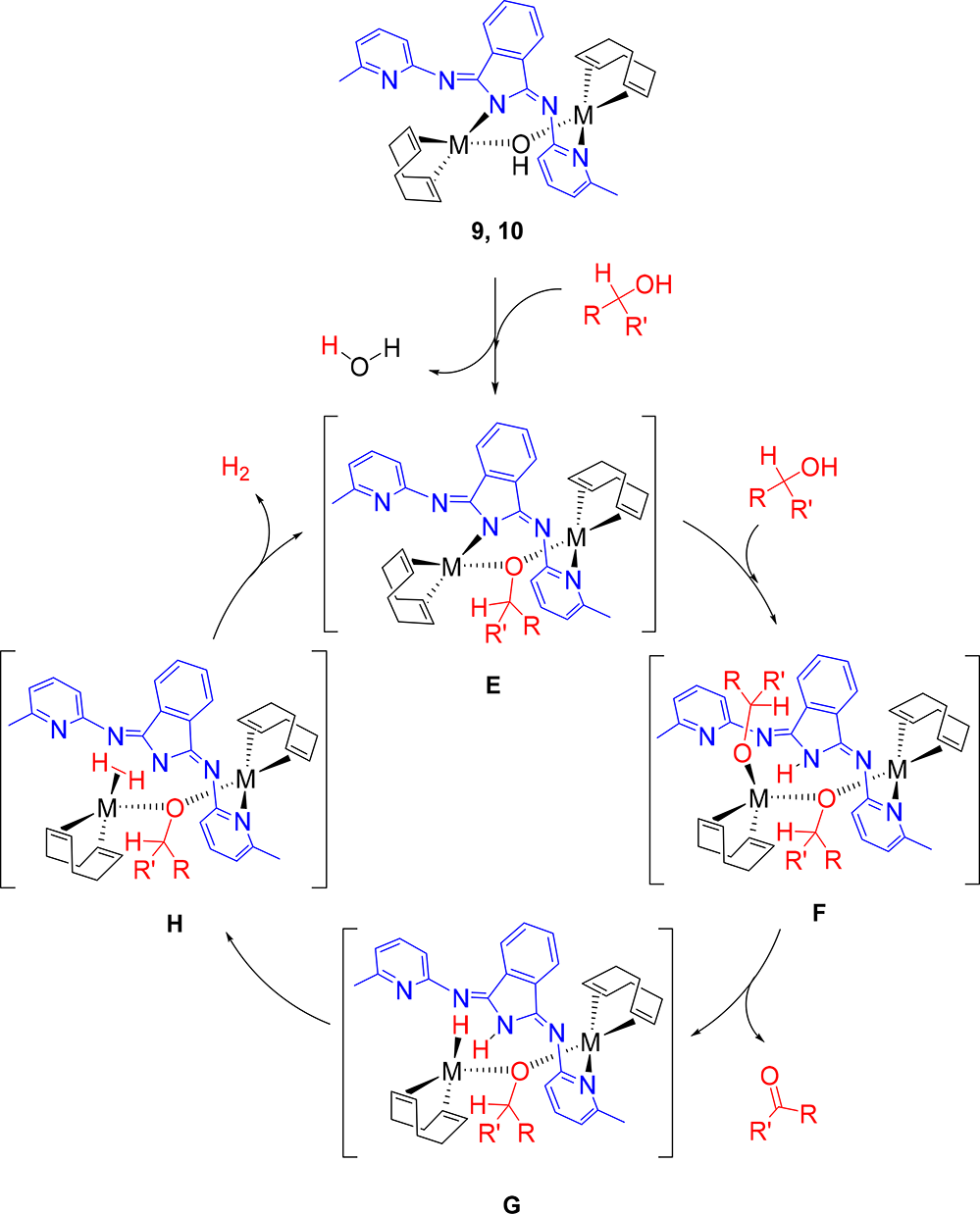
Catalytic Cycle for the Acceptorless and Base-Free Dehydrogenation of Secondary Alcohols Promoted by Complexes **9** and **10**

The catalysis can be rationalized according to Scheme 8. The alcohol, which is in great excess with regard to the metal complexes, should initially displace the bridging hydroxide ligand to afford the related alkoxide derivatives [M(η^4^-C_8_H_12_)]_2_(μ-OCHRR′){μ-*N*_iso_,*N*_py_-(BMePHI)} (**E**), which would be the catalytically active species of the dehydrogenation process. As in the reactions catalyzed by the polyhydrides shown in Scheme 1,^10^ the addition of the O–H bond of the alcohols to the bond M–*N*_iso_ of **E** could generate the intermediates (η^4^-C_8_H_12_)(R′RCHO)M(μ-OCHR′R)M{κ^1^-*N*_py_-(HBMePHI)}(η^4^-C_8_H_12_) (**F**), the alkoxide counterparts of **A**–**D**. Then, the subsequent β-hydrogen elimination on the terminal alkoxide group could afford the ketone and the hydride species (η^4^-C_8_H_12_)HM(μ-OCHR′R)M{κ^1^-*N*_py_-(HBMePHI)}(η^4^-C_8_H_12_) (**G**), which would evolve into (η^4^-C_8_H_12_)(η^2^-H_2_)M(μ-OCHR′R)M{κ^1^-*N*_py_-(BMePHI)}(η^4^-C_8_H_12_) (**H**) via heterolytic H–H formation.^31^ The subsequent substitution of the dihydrogen ligand by the isoindoline-N atom should release molecular hydrogen, regenerating the active species **E**.

The cycle shown in Scheme 8 is consistent with those proposed for the dehydrogenations promoted by the polyhydrides of Scheme 1. The noninnocent character of the bridging ligand is shown by the addition of the O–H bond of the alcohol to the M–*N*_iso_ bond of **E** and in the formation of **H**. However, in this case, only a metal center would have a direct participation in the catalysis. The function of the other should be to keep the isoindoline N–H bond in the proximity of the active center.

## Concluding Remarks

This study has revealed that a pyridyl group of 1,3-bis(6′-methyl-2′-pyridylimino)isoindoline (HBMePHI) coordinates to a metal center of the dimers [M(μ-X)(η^4^-C_8_H_12_)]_2_ (M = Rh, Ir; X = Cl, OH) to afford square-planar species, splitting at least one of the bridges. The subsequent deprotonation of the N–H bond of the isoindoline core by a hydroxo group leads to the complexes [M(η^4^-C_8_H_12_)]_2_(μ-OH){μ-*N*_iso_,*N*_py_-(BMePHI)} (M = Rh, Ir), which are efficient catalyst precursors for the acceptorless and base-free dehydrogenation of secondary alcohols. These compounds cannot be used in catalytic processes needing a basic medium, because the [BMePHI]^−^ ligand undergoes degradation. Depending upon the metal center of the complex, three different deteriorations have been observed: (i) alcoholysis of an exocyclic isoindoline-N double bond, (ii) alcoholysis of a N-pyridyl bond, and (iii) opening of the five-membered ring of the isoindoline core by oxidative addition of one of the C–N bonds to a metal center of the catalyst precursor.

In summary, 1,3-bis(2′-pyridylimino)isoindolinates are interesting organic anions, which can act as noninnocent bridging ligands in diverse catalysts for the acceptorless and base-free dehydrogenation of secondary alcohols. However, they should be not employed to stabilize catalysts of processes which take place in basic media, since they undergo degradation.

## Experimental Section

All reactions were carried out with rigorous exclusion of air using Schlenk-tube techniques. Solvents were dried using standard procedures and distilled under an argon atmosphere or obtained dry from an MBraun solvent purification apparatus. ^1^H and ^13^C{^1^H} NMR spectra (Figures S10–S30) were recorded on a Bruker Avance 300 MHz, Bruker Avance 400 MHz, or Bruker Avance 500 MHz instrument. Chemical shifts (expressed in ppm) are referenced to residual solvent peaks (^1^H, ^13^C{^1^H}). Coupling constants *J* are given in hertz. C, H, and N analyses were carried out with a PerkinElmer 2400 CHNS/O analyzer or with a Thermo FlashEA 1112 CHNS/O analyzer. High-resolution electrospray mass spectra (HRMS) were acquired using a MicroTOF-Q hybrid quadrupole time-of-flight spectrometer (Bruker Daltonics, Bremen, Germany). [Rh(μ-Cl)(η^4^-C_8_H_12_)]_2_ (**1**),^32^ [Ir(μ-Cl)(η^4^-C_8_H_12_)]_2_ (**2**),^33^ [Rh(μ-OH)(η^4^-C_8_H_12_)]_2,_ (**3**),^34^ [Ir(μ-OH)(η^4^-C_8_H_12_)]_2_ (**4**),^35^ and 1,3-bis(6′-methyl-2′-pyridylimino)isoindoline (HBMePHI)^36^ were prepared according to the published methods.

### General Procedure for the Rh- and Ir-Catalyzed Dehydrogenation Reactions of Alcohols

A solution of the catalyst (**9** or **10**, 0.009 mmol) and the corresponding substrate (0.255 mmol) in toluene (1 mL) was placed in a Schlenk flask equipped with a condenser under an argon atmosphere. The mixture was stirred at 100 °C for 24 h. After this time the solution was cooled to room temperature, and the progress of the reaction was monitored by GC (Agilent 6890N gas chromatograph with a flame ionization detector, using an Agilent 19091N-133 polyethylene glycol column (30 m × 250 μm × 0.25 μm thickness)). The oven conditions used are as follows: 80 °C (hold 5 min) to 200 °C at 15 °C/min (hold 7 min), except for diphenylmethanol, 150 °C (hold 5 min) to 240 °C at 15 °C/min (hold 13 min). The obtained values of the yield are the average of two runs. The identity of the compound was confirmed by comparison of the retention time of the product.

### Addition of 2.0 mol of HBMePHI to [Rh(μ-Cl)(η^4^-C_8_H_12_)]_2_ (**1**)

At room temperature, 19 mg (0.06 mmol) of HBMePHI was added to 0.5 mL of a dichloromethane-*d*_2_ solution of **1** (15 mg, 0.03 mmol) in an NMR tube. After 10 min, ^1^H NMR spectra between 293 and 223 K (Figure 1) and the ^13^C{^1^H} NMR spectrum at 213 K (Figure S12) were recorded. HRMS (electrospray, *m*/*z*): calcd for C_28_H_29_RhN_5_ClNa [M + Na]^+^ 596.1059, found 596.1038. Selected spectroscopic data for RhCl(η^4^-C_8_H_12_){κ^1^-*N*_py_-(HBMePHI)} (**5**) are as follows. ^1^H NMR (400 MHz, CD_2_Cl_2_, 223 K): δ 12.70 (s, 1H, NH), 8.91 (d, ^3^*J*_H–H_ = 7.4, 1H, CH_arom_), 8.04 (dt, ^3^*J*_H–H_ = 7.4, ^3^*J*_H–H_ = 1.0, 1H, CH_arom_), 7.86 (td, ^3^*J*_H–H_ = 7.5, ^3^*J*_H–H_ = 1.2, 1H, CH_arom_), 7.80 (td, ^3^*J*_H–H_ = 7.5, ^3^*J*_H–H_ = 1.2, 1H, CH_arom_), 7.74 (t, ^3^*J*_H–H_ = 7.8, 1H, CH_arom_), 7.63 (t, ^3^*J*_H–H_ = 7.6, 1H, CH_arom_), 7.25 (d, ^3^*J*_H–H_ = 7.9, 1H, CH_arom_), 7.21 (d, ^3^*J*_H–H_ = 7.9, 1H, CH_arom_), 7.08 (d, ^3^*J*_H–H_ = 7.6, 1H, CH_arom_), 6.92 (d, ^3^*J*_H–H_ = 7.5, 1H, CH_arom_), 4.46 (m, 1H, CH COD), 4.38 (m, 1H, CH COD), 3.55 (m, 1H, CH COD), 3.50 (m, 1H, CH COD), 3.17 (s, 3H, CH_3_), 2.36 (m, 3H, CH_2_ COD), 2.24 (s, 3H, CH_3_), 1.98 (m, 1H, CH_2_ COD), 1.70 (m, 2H, CH_2_ COD), 1.66 (m, 1H, CH_2_ COD), 1.46 (m, 1H, CH_2_ COD). ^13^C-APT NMR (100.6 MHz, CD_2_Cl_2_, 213 K): δ 159.5, 159.4, 159.0, 156.0, 153.0, 152.9 (all s, C_arom_), 139.2, 138.7 (both s, CH_arom_), 135.8, 133.3 (both s, C_arom_), 132.6, 132.0, 123.6, 122.2, 121.0, 120.3, 120.2, 113.7 (all s, CH_arom_), 81.7 (d, ^2^*J*_C–Rh_ = 12.2, CH COD), 80.4 (d, ^2^*J*_C–Rh_ = 11.5, CH COD), 75.6 (d, ^2^*J*_C–Rh_ = 13.6, CH COD), 74.6 (d, ^2^*J*_C–Rh_ = 13.4, CH COD), 31.4, 30.3, 30.0, 29.9 (all s, CH_2_ COD), 25.5, 24.2 (both s, py-CH_3_).

### Addition of 1.0 mol of HBMePHI to [Rh(μ-Cl)(η^4^-C_8_H_12_)]_2_

At room temperature, 10 mg (0.03 mmol) of HBMePHI was added to 0.5 mL of a dichloromethane-*d*_2_ solution of **1** (15 mg, 0.03 mmol) in an NMR tube. After 10 min, ^1^H NMR spectra between 283 and 183 K (Figure S14) and the ^13^C{^1^H} NMR spectrum at 183 K (Figure S15) were recorded. HRMS (electrospray, *m*/*z*): calcd for C_36_H_41_Rh_2_N_5_Cl [M – Cl]^+^ 784.1155, found 784.1190. Selected spectroscopic data for RhCl(η^4^-C_8_H_12_){μ-*N*_py_,*N*_py_-(HBMePHI)}(**6**): are as follows. ^1^H NMR (400 MHz, CD_2_Cl_2_, 183 K): δ 8.1–8.0 (3H, CH_arom_), 7.8–7.7 (4H, CH_arom_), 7.1–7.0 (3H, CH_arom_), 4.50 (br, 1H, CH COD), 4.44 (br, 1H, CH COD), 3.48 (br, 1H, CH COD), 3.42 (br, 1H, CH COD), 2.80 (s, 6H, py-CH_3_), 2.40 (m, 4H, CH_2_ COD), 2.33 (m, 4H, CH_2_ COD), 1.66 (m, 8H, CH_2_ COD). ^13^C{^1^H}-APT NMR (100.6 MHz, CD_2_Cl_2_, 193 K): δ 158.6, 158.4, 157.5 (all s, C_arom_), 139.1 (s, 2C, CH_arom_), 133.7 (s, C_arom_), 132.7, 121.1 (s, 2C, CH_arom_), 81.8, 80.8, 76.1, 75.7 (all br, CH COD), 30.8, 30.2, 29.9, 29.6 (all s, CH_2_ COD), 24.9 (s, 2C, py–CH_3_).

### Preparation of IrCl(η^4^-C_8_H_12_){κ^1^-*N*_py_-(HBMePHI)} (**7**)

Complex **2** (0.300 g, 0.446 mmol) was dissolved in toluene (6 mL) and treated with 2.0 mol of HBMePHI (0.320 g, 0.981 mmol). The resulting suspension turned red and was stirred for 2 h. Then, the volatiles were removed under vacuum. The residue was washed with pentane (3 × 3 mL) to afford a red solid, which was dried *in vacuo*. Yield: 323 mg (54%). Red crystals suitable for X-ray diffraction analysis were obtained from slow diffusion of pentane in a concentrated solution of **7** in toluene. Anal. Calcd for C_28_H_29_ClIrN_5_: C, 50.71; H, 4.41; N, 10.56. Found: C, 50.37; H, 4.59; N, 10.55. HRMS (electrospray, *m*/*z*): calcd for for C_28_H_29_IrN_5_ [M – Cl]^+^ 628.2046, found 628.2047. ^1^H NMR (400 MHz, CD_2_Cl_2_, 243 K): δ 12.72 (s, 1 H, NH), 8.61 (dd, ^3^*J*_H–H_ = 6.2, ^3^*J*_H–H_ = 1.6, CH_iso_), 8.03 (dd, ^3^*J*_H–H_ = 6.2, ^3^*J*_H–H_ = 1.6, 1H, CH_iso_), 7.81–7.80 (m, 2H, CH_arom_), 7.76 (dd, ^3^*J*_H–H_ = 7.6, ^3^*J*_H–H_ = 7.6, 1H, CH_py_), 7.66 (m, 1H, CH_py_), 7.22 (d, 1H, ^3^*J*_H–H_ = 8.3, CH_py_) 7.20 (d, 1H, ^3^*J*_H–H_ = 8.3, CH_py_), 7.13 (d, 1H, ^3^*J*_H–H_ = 7.6, CH_py_), 6.94 (d, 1 H, ^3^*J*_H–H_ = 7.6, CH_py_), 4.23 (m, 1H, = CH COD), 4.10 (m, 1H, = CH COD), 3.22 (m, 1 H, = CH COD), 3.12 (m, 1 H, = CH COD), 3.03 (s, 3 H, CH_3_), 2.27 (s, 3 H, CH_3_), 2.15 (m, 3 H, CH_2_ COD), 1.99 (m, 1 H, CH_2_ COD), 1.72 (m, 1 H, CH_2_ COD), 1.38 (m, 4 H, COD), 1.12 (m, 1 H, COD). ^13^C{^1^H}-APT NMR (plus HSQC and HMBC) (100.6 MHz, CD_2_Cl_2_, 243 K): δ 159.6, 159.5, 159.4, 156.3 (all s, C_py_), 153.2, 152.5 (both s, C_iso_) 139.2, 139.0 (both s, CH_py_) 136.1, 133.5 (both s, C_iso_) 132.7, 132.3, 124.0, 122.5 (all s, CH_iso_), 121.8, 120.6, 120.4, 114.6 (all s, CH_py_) 66.3, 64.3, 58.1, 57.4 (all s, =CH COD) 32.2, 31.3 (both s, CH_2_ COD) 31.0 (s, 2 C, CH_2_ COD), 25.3, 24.4 (both s, py-CH_3_).

### Preparation of {IrCl(η^4^-C_8_H_12_)}_2_(μ-*N*_py_,*N*_py_-HBMePHI) (**8**)

Complex **2** (0.300 g, 0.446 mmol) was dissolved in toluene (8 mL) and treated with 1.0 mol of HBMePHI (0.145 g, 0.446 mmol). The orange suspension turned red, and a yellow precipitate was formed. After 4 h, at room temperature, the solid was separated from the liquors. The yellow solid was washed with pentane (3 × 3 mL, 273 K) and was dried *in vacuo*. Yield: 357 mg (80%). Yellow crystals suitable for X-ray diffraction analysis were obtained from slow diffusion of diethyl ether in a concentrated solution of **8** in dichloromethane. Anal. Calcd for C_36_H_41_Cl_2_Ir_2_N_5_: C, 43.28; H, 4.14; N, 7.01. Found: C, 42.88; H, 3.79; N, 7.34. HRMS (electrospray, *m*/*z*): calcd for C_36_H_41_Ir_2_N_5_Cl [M −Cl]^+^ 964.2304, found 964.2286. ^1^H NMR (400 MHz, CD_2_Cl_2_, 253 K): δ 12.70 (s, 1H, NH), 8.83 (m, 2H, CH_arom_), 7.92 (m, 2H, CH_arom_), 7.62 (m, 2H, CH_arom_), 7.33 (d, 2H, ^3^*J*_H–H_ = 8.1, CH_arom_), 7.02 (d, 2H, ^3^*J*_H–H_ = 8.1, CH_arom_), 4.25 (m, 2H, CH COD), 4.05 (m, 2H, CH COD), 3.58 (m, 2H, CH COD), 3.25 (m, 2H, CH COD), 2.85 (s, 6H, py-CH_3_), 2.19 (m, 2H, CH_2_ COD), 1.99 (m, 2H, CH_2_ COD), 1.51 (m, 2H, CH_2_ COD), 1.41 (m, 2H, CH_2_ COD). ^13^C{^1^H}-APT NMR (100.6 MHz, CD_2_Cl_2_, 253 K): δ 159.6, 158.2, 152.6 (all s, C_arom_) 139.4 (s, CH_arom_), 134.6 (s, C_arom_), 133.0, 124.6, 121.9, 116.4 (all s, CH_arom_), 66.1, 65.8, 59.1, 58.6 (all s, =CH COD), 32.4, 31.9, 31.3, 30.8 (all s, CH_2_ COD), 25.2 (s, py–CH_3_).

### Preparation of [Rh(η^4^-C_8_H_12_)]_2_(μ-OH){μ-*N*_iso_,*N*_py_-(BMePHI)} (**9**)

The substrate HBMePHI (0.071 g, 0.219 mmol) was added to 3 mL of a propan-2-ol suspension of **3** (0.1 g, 0.219 mmol). After 2 h an orange solid was formed. The liquors were separated, and the orange solid was washed with 2 mL of propan-2-ol at 0 °C. The solid was solved in toluene. The solution was concentrated *in vacuo*. The addition of pentane gives rises to the precipitation of an orange solid which was washed with pentane (2 × 3 mL) at 0 °C. Yield: 132 mg (80%). Orange crystals of **9** were obtained from slow diffusion of pentane in toluene. Anal. Calcd for C_36_H_41_N_5_ORh_2_: C, 56.48; H, 5.40; N, 9.15. Found: C, 56.20; H, 5.35; N, 9.28. HRMS (electrospray, *m*/*z*): calcd for for C_36_H_40_N_5_Rh_2_ [M – OH]^+^ 748.1388, found 748.1392. ^1^H NMR (400 MHz, toluene-*d*_8_, 183 K): δ 8.43 (dd, ^3^*J*_H–H_ = 4.1, 3.0, 1H, CH_arom_), 8.31 (dd, ^3^*J*_H–H_ = 4.1, 3.0, 1H, CH_arom_), 7.30 (d, ^3^*J*_H–H_ = 7.8, 1H, CH_arom_), 7.22 (m, 1H, CH_arom_), 7.14 (m, 1H, CH_arom_), 7.07 (m, 1H, CH_arom_), 6.98 (d, ^3^*J*_H–H_ = 7.8, 1H, CH_arom_), 6.88 (dd, ^3^*J*_H–H_ = 7.8, 7.8, 1H, CH_arom_), 6.41 (d, ^3^*J*_H–H_ = 7.5, 1H, CH_arom_), 6.22 (d, ^3^*J*_H–H_ = 7.5, 1H, CH_arom_), 5.34 (m, 1H, CH COD), 4.31 (m, 1H, CH COD), 4.02 (m, 1H, CH COD), 3.64 (m, 1H, CH COD), 3.48 (m, 1H, CH COD), 3.36 (m, 1H, CH COD), 3.27 (m, 1H, CH COD), 3.21 (m, 1H, CH COD), 3.18 (s, 3H, py–CH_3_), 2.44 (m, 2H, CH_2_ COD), 2.40 (s, 3H, py-CH_3_), 2.22 (s, 1H, Rh–OH–Rh), 1.86 (m, 2H, CH_2_ COD), 1.74 (m, 2H, CH_2_ COD), 1.56 (m, 2H, CH_2_ COD), 1.31 (m, 2H, CH_2_ COD), 1.26 (m, 2H, CH_2_ COD), 1.12 (m, 2H, CH_2_ COD), 1.01 (m, 2H, CH_2_ COD). ^13^C{^1^H}-APT NMR (100.6 MHz, toluene-*d*_8_, 193 K): δ 166.5. 165.1, 164.2, 161.0, 158.6, 156.6, 141.9, 140.9 (all s, C_arom_), 138.7, 138.4, 131.0, 130.6, 122.3, 121.6, 118.0, 117.9, 117.4, 116.7 (all s, CH_arom_), 83.7, 81.8, 81.0, 79.3, 74.7, 72.9, 71.5, 71.3 (all s, CH COD), 34.2 (s, CH_2_ COD), 31.7 (s, 2C, CH_2_ COD), 31.2, 30.1, 29.9, 29.3, 28.9 (all s, CH_2_ COD), 26.5 (py-CH_3_), 25.3 (py–CH_3_).

### Preparation of [Ir(η^4^-C_8_H_12_)]_2_(μ-OH){μ-*N*_iso_,*N*_py_-(BMePHI)} (**10**)

The substrate HBMePHI (0.051 g, 0.157 mmol) was added to 3 mL of a propan-2-ol suspension of **4** (0.1 g, 0.157 mmol). After 2 h an orange solid was formed. The liquors were separated, and the orange solid was washed with 2 mL of propan-2-ol at 0 °C. The solid was solved in toluene. The solution was concentrated *in vacuo*. The addition of pentane gives rise to the precipitation of an orange solid, which was washed with pentane (2 × 3 mL) at 0 °C. Yield: 70 mg (47%). Orange crystals of **10** were obtained from slow diffusion of pentane in toluene. Anal. Calcd for C_36_H_41_Ir_2_N_5_O: C, 45.80; H, 4.38; N, 7.42. Found: C, 46.10; H, 4.26, N, 7.52. HRMS (electrospray, *m*/*z*): calcd for C_36_H_40_Ir_2_N_5_ [M – OH]^+^ 928.2537, found 928.2585. ^1^H NMR (400 MHz, toluene-*d*_8_, 193 K): δ 8.28 (br, 1H, CH_arom_), 8.20 (br, 1H, CH_arom_), 7.13 (br, 3H, CH_arom_), 7.08 (br, 1H, CH_arom_), 6.90 (br, 1H, CH_arom_), 6.77 (br, 1H, CH_arom_), 6.32 (br, 1H, CH_arom_), 6.09 (br, 1H, CH_arom_), 5.07 (br, 1H, CH COD), 4.24 (br, 1H, Ir–OH–Ir), 4.05 (br, 1H, CH COD), 3.86 (br, 1H, CH COD), 3.44 (br, 1H, CH COD), 3.20 (br, 3H, CH COD), 2.99 (br, 1H, CH COD), 2.87 (s, 3H, py–CH_3_), 2.33 (s, 3H, py-CH_3_), 2.32 (br, 2H, CH_2_ COD), 1.86 (br, 2H, CH_2_ COD), 1.68 (br, 2H, CH_2_ COD), 1.44 (br, 2H, CH_2_ COD), 1.36 (br, 2H, CH_2_ COD), 1.09 (br, 2H, CH_2_ COD), 0.98 (br, 2H, CH_2_ COD), 0.82 (br, 2H, CH_2_ COD). ^13^C{^1^H}-APT NMR (plus HSQC and HMBC) (100.6 MHz, toluene-*d*_8_, 188 K): δ 165.4, 164.9, 163.8, 159.2, 157.0, 155.7, 140.8, 139.9 (all s, C_arom_), 138.1, 138.0, 130.7, 130.3, 122.0, 121.4, 118.2, 118.0, 116.9, 116.3 (all s, CH_arom_), 67.4, 66.5, 65.6, 62.4, 56.3, 55.2, 53.0, 52.2 (all s, CH COD), 34.6, 32.3, 32.2, 31.7, 30.7, 30.2, 30.1, 29.0 (all s, CH_2_ COD), 25.6, 24.7 (both s, py-CH_3_).

### Preparation of [Rh(η^4^-C_8_H_12_)]_2_{μ-*N*_iso_,*N*_imine_-(HN=C_8_H_4_NO)}(μ-N=C_8_H_4_NO^*i*^Pr) (**11**)

The substrate HBMePHI (173.2 mg, 0.531 mmol) and KO^t^Bu (89 mg, 0.796 mmol) were added to 3 mL of a propan-2-ol suspension of **3** (121 mg, 0.265 mmol). The mixture was stirred for 2 h at room temperature, and an orange solid was formed, which was filtered off. The filtrate was cooled at 4 °C for 3 days, and red crystals were obtained. Yield: 40 mg (20%). Anal. Calcd for C_35_H_40_N_4_O_2_Rh_2_: C, 55.71; H, 5.34; N, 7.43. Found: C, 55.87; H, 5.42; N, 7.38. HRMS (electrospray, *m*/*z*): calcd for C_35_H_41_N_4_O_2_Rh_2_ [M + H]^+^ 755.1334, found 755.1308. ^1^H NMR (400 MHz, C_6_D_6_, 298 K): δ 10.61 (d, ^3^*J*_H–H_ = 7.5, 1 H, CH_arom_), 7.65 (t, ^3^*J*_H–H_ = 7.5, 1 H, CH_arom_), 7.39 (d, ^3^*J*_H–H_ = 7.3, 1 H, CH_arom_), 7.27 (d, ^3^*J*_H–H_ = 7.3, 1 H, CH_arom_), 7.02 (dd, ^3^*J*_H–H_ = 7.4, 7.2, 1 H, CH_arom_), 6.74 (dd, ^3^*J*_H–H_ = 7.4, 7.2, 1 H, CH_arom_), 6.64 (dd, ^3^*J*_H–H_ = 7.4, 7.2, 1 H, CH_arom_), 6.44 (m, 1 H, CH COD), 6.25 (d, ^3^*J*_H–H_ = 7.5, 1 H, CH_arom_), 6.21 (m, 1 H, CH COD), 5.78 (br, 1 H, NH), 5.36 (sept, ^3^*J*_H–H_ = 6.4, 1H, OC*H*(CH_3_)_2_), 5.15 (m, 1 H, CH COD), 4.98 (dd, ^3^*J*_H–H_ = 7.2, 6.9, 1 H, CH COD), 4.54 (dd, ^3^*J*_H–H_ = 7.2, 6.9, 1 H, CH COD), 3.96 (dd, ^3^*J*_H–H_ = 7.2, 6.9, 1 H, CH COD), 3.55 (m, 1 H, CH COD), 3.44 (m, 1 H, CH COD), 3.23 (m, 2 H, CH_2_ COD), 3.11 (m, 2 H, CH_2_ COD), 2.36 (m, 6 H, CH_2_ COD), 2.16 (m, 2 H, CH_2_ COD), 1.67 (m, 4 H, CH_2_ COD), 1.26 and 1.15 (both d, ^3^*J*_H–H_ = 6.4, 3 H each, OCH(C*H*_3_)_2_). ^13^C{^1^H}-APT NMR (plus HSQC and HMBC) (100.6 MHz, CD_2_Cl_2_, 298 K): δ 176.8, 175.3, 174.5, 163.8, 136.5, 136.0, 134.9, 134.6 (all s, C_arom_), 130.8, 130.6, 129.8, 128.8, 125.9, 121.9, 119.1, 118.4 (all s, CH_arom_), 86.9 (d, ^2^*J*_C–Rh_ = 12.9, CH COD), 84.2 (d, ^2^*J*_C–Rh_ = 8.9, CH COD), 81.9 (d, ^2^*J*_C–Rh_ = 11.7, CH COD), 81.6 (d, ^2^*J*_C–Rh_ = 10.5, CH COD) 81.2 (d, ^2^*J*_C–Rh_ = 10.5, CH COD), 79.0 (d, ^2^*J*_C–Rh_ = 10.5, CH COD), 78.2 (d, ^2^*J*_C–Rh_ = 12.9, CH COD), 74.5 (d, ^2^*J*_C–Rh_ = 11.7, CH COD), 72.2 (s, O*C*H(CH_3_)_2_), 35.2, 35.2, 35.0, 34.3, 29.5, 29.0, 28.6, 28.5 (all s, CH_2_ COD), 22.0 and 21.9 (both s, OCH*C*H_3_).

### Preparation of Ir_3_(η^4^-C_8_H_12_)_2_(κ^1^-*C*,η^2^-C_8_H_13_)(μ-OH)(L) (**12**)

The substrate HBMePHI (147 mg, 0.450 mmol) and KO^t^Bu (75 mg, 0.675 mmol) were added to 4 mL of a propan-2-ol suspension of **4** (142 mg, 0.225 mmol). The mixture was stirred for 2 h at room temperature, and an orange solid was formed, which was filtered off and subsequently was treated with toluene (2 × 4 mL) to afford a yellow solid and a red solution. The solution was separated by filtration, and the volatiles were removed under vacuum. The treatment of the residue with pentane gave an orange solid. Orange crystals suitable for X-ray diffraction analysis were obtained from a saturated solution in benzene. Yield: 42 mg (22%). Anal. Calcd for C_44_H_54_Ir_3_N_5_O: C, 42.43; H, 4.37; N, 5.62. Found: C, 42.18; H, 4.38; N, 5.39. HRMS (electrospray, *m*/*z*): calculated for C_44_H_53_Ir_3_N_5_ [M – OH]^+^ 1230.3183, found 1230.3152. ^1^H NMR (500.13 MHz, C_6_D_6_, 298 K): δ 8.53 (dd, ^3^*J*_H–H_ = 7.8, 1.0, 1H, H^12^), 8.07 (dd, ^3^*J*_H–H_ = 7.3, 1.4, 1H, H^9^), 7.64 (dd, ^3^*J*_H–H_ = 7.9, 1.0, 1H, CH_py_), 7.29 (m, 2H, CH_arom_), 7.25 (dd, ^3^*J*_H–H_ = 7.4, 1.4, 1H, H^11^), 7.14 (m, 1H, CH_py_), 7.13 (m, 1H, CH_py_), 6.69 (dd, ^3^*J*_H–H_ = 7.3, 1.0, 1H, CH_py_), 5.94 (d, ^3^*J*_H–H_ = 7.2, 1H, CH_py_), 5.55 (m, 1H, CH COD), 4.54 (m, 1H, CH COD), 4.12 (m, 1H, CH COD), 4.04 (m, 1H, CH COD), 3.88 (m, 1H, CH COD), 3.82 (m, 1H, CH COD), 3.69 (m, 1H, CH COD), 3.46 (m, 1H, CH COD), 3.37 (s, 3H, py-CH_3_), 3.11 (m, 2H, CH COD), 2.58 (m, 1H, CH_2_ COD), 2.50 (m, 1H, CH_2_ COD), 2.32 (m, 1H, CH_2_ COD), 2.29 (m, 1H, CH_2_ COD), 2,13 (m, 3H, CH_2_ COD), 2.04 (m, 3H, CH_2_ COD), 1.94 (m, 4H, CH_2_ COD), 1.85 (s, 3H, py-CH_3_), 1.79 (m, 1H, CH_2_ COD), 1.75 (m, 1H, CH_2_ COD), 1.20 (m, 3H, CH_2_ COD), 1.11 (m, 2H, CH_2_ COD), 0.65 (m, 1H, κ^1^-CH COD), 0.24 (s, 1H, Ir–OH–Ir). ^13^C{^1^H}-APT NMR (plus HSQC and HMBC) (75 MHz, C_6_D_6_, 298 K): δ 198.9 (s, C^7^), 179.3 (s, C_py_), 166.7 (s, C_py_), 159.4 (s, C^14^), 157.9 (s, C_py_), 153.1 (s, C_py_), 145.8 (s, C^13^), 137.7 (s, C_py_), 137.6 (s, C_py_), 135.4 (s, C^8^), 129.1 (s, C^12^), 128.8 (s, C^11^), 127.9 (s, C^10^), 122.3 (s, C^9^), 119.6 (s, C_py_), 117.6 (s, C_py_), 111.1 (s, C_py_), 110.3 (s, C_py_), 86.4, 71.4, 67.7, 67.0, 63.0, 60.1, 59.7, 59.0, 59.0, 52.6 (all s, = CH COD), 42.7, 36.9, 35.8, 34.5, 32.6, 32.5 (all s, CH_2_ COD), 32.1 (κ^1^-CH COD), 31.1, 30.7, 30.3, 29.5, 28.5, 28.2 (all s, CH_2_ COD), 25.0 (s, py-CH_3_), 23.4 (s, CH_2_ COD), 21.0 (s, py-CH_3_).

## Supporting Information Available

Computational and electrochemical data, structural analysis of complexes **7**–**9**, **11**, and **12**, and NMR spectra (PDF)
Cartesian coordinates of computed complexes (XYZ)

## Supplementary Material

om1c00068_si_001.pdf

om1c00068_si_002.xyz
