# Supplementary material for: Preparation and Degradation of Rhodium and Iridium Diolefin Catalysts for the Acceptorless and Base-Free Dehydrogenation of Secondary Alcohols
Source: Organometallics. 2021 Mar 31;40(7):989–1003. doi: 10.1021/acs.organomet.1c00068 (PMC9180741; doi:10.1021/acs.organomet.1c00068)
Supplement: Supplementary file 1 — om1c00068_si_001.pdf [file om1c00068_si_001.pdf]

Supporting Information

Preparation and Degradation of Rhodium- and Iridium-Diolefin Catalysts for the Acceptorless and Base-Free Dehydrogenation of Secondary Alcohols

María L. Buil,[a] Alba Collado,[b] Miguel A. Esteruelas,[,a] Mar Gómez-Gallego,[b] Susana Izquierdo,[a] Antonio I. Nicasio,[a] Enrique Oñate,[a] and Miguel A. Sierra.*[,b]*

[a]Departamento de Química Inorgánica, Instituto de Síntesis Química y Catálisis Homogénea (ISQCH), Centro de Innovación en Química Avanzada (ORFEO-CINQA), Universidad de Zaragoza-CSIC, 50009 Zaragoza, Spain

[b]Departamento de Química Orgánica I, Facultad de CC. Químicas, Centro de Innovación en Química Avanzada (ORFEO-CINQA) Universidad Complutense de Madrid, 28040 Madrid, Spain

*Corresponding author's e-mail address: M.A.E.: maester@unizar.es;
M.A.S.: sierraor@quim.ucm.es

Contents:

Structural Analysis of Complexes 7, 8, 9, 11 and 12 **S3**

Electrochemical Data **S5**

Computational Data **S8**

^{1}H NMR spectrum, ^{1}H NMR spectra as a function of the temperature and ^{13}C{^{1}H} spectrum of the solution resulting of the addition of 2.0 mol of HBMePHI to 1 (Figures S10 – S12) **S13**

^{1}H NMR spectrum, ^{1}H NMR spectra as a function of the temperature and ^{13}C{^{1}H} spectrum of the solution resulting of the addition of 1.0 mol of HBMePHI to 1 (Figures S13 – S15) **S14**

^{1}H NMR spectrum, ^{1}H NMR spectra as a function of the temperature and ^{13}C{^{1}H} spectrum of complex 7 (Figures S16 – S18) **S16**

^{1}H NMR spectra as a function of the temperature and ^{13}C{^{1}H} spectrum of complex 8 (Figures S19 – S20) S17

^{1}H NMR spectrum, ^{1}H NMR spectra as a function of the temperature, ^{13}C{^{1}H} spectrum of complex 9 (Figures S21 – S23) S18

^{1}H NMR spectrum, ^{1}H NMR spectra as a function of the temperature, ^{13}C{^{1}H} spectrum of complex 10 (Figures S24 – S26) S20

^{1}H NMR spectrum and ^{13}C{^{1}H} spectrum of complex 11 (Figures S27 – S28) S21

^{1}H NMR spectrum and ^{13}C{^{1}H} spectrum of complex 12 (Figures S29 – S30) S22

References S24

Structural Analysis of Complexes 7, 8, 9, 11 and 12

X-ray data were collected for the complexes on a Bruker Smart APEX or Bruker Smart APEX DUO CCD diffractometers equipped with a normal focus, and 2.4 kW sealed tube source (Mo radiation, λ = 0.71073 Å). Data were collected over the complete sphere covering 0.3^o in ω. Data were corrected for absorption by using a multiscan method applied with the SADABS program.[1] The structures were solved by Patterson or direct methods and refined by full-matrix least squares on F^2 with SHELXL2016,[2] including isotropic and subsequently anisotropic displacement parameters. The hydrogen atoms were observed in the last Fourier Maps or calculated, and refined freely or using a restricted riding model.

Crystal data for **7**: $C_{28}H_{29}ClIrN_5$, M_W 663.21, red, irregular block (0.180 x 0.060 x 0.030 mm^3), monoclinic, space group $P2_1/n$, *a*: 14.1757(11) Å, *b*: 7.8994(6) Å, *c*: 22.1415(17) Å, *β*: 99.9390(10)°, *V* = 2442.2(3) Å^3, *Z* = 4, *Z'* = 1, D_{calc}: 1.804 g cm^{-3}, F(000): 1304, T = 100(2) K, μ 5.604 mm^{-1}. 31070 measured reflections (2θ: 3-57°, ω scans 0.3^o), 5954 unique (R_{int} = 0.0611); min./max. transm. Factors 0.636/0.746. Final agreement factors were R^1 = 0.0347 (4588 observed reflections, I > 2σ(I)) and wR^2 = 0.0743; data/restraints/parameters 5954/0/333; GoF = 1.023. Largest peak and hole 2.734 (close to iridium atoms) and -1.148 e/ Å^3.

Crystal data for **8**: $C_{36}H_{41}Cl_2Ir_2N_5$, M_W 999.04, red, irregular block (0.275 x 0.075 x 0.056 mm^3), monoclinic, space group C2/c, *a*: 28.035(7) Å, *b*: 9.735(2) Å, *c*: 12.963(3) Å, *β*: 106.789(3)°, *V* = 3387.0(14) Å^3, *Z* = 4, *Z'* = 0.5, D_{calc}: 1.959 g cm^{-3}, F(000): 1920, T = 100(2) K, μ 8.042 mm^{-1}. 13485 measured reflections (2θ: 3-57°, ω scans 0.3^o), 4019 unique (R_{int} = 0.0740); min./max. transm. Factors 0.431/0.862. Final agreement factors

were R^1 = 0.0447 (2875 observed reflections, I > 2σ(I)) and wR^2 = 0.1119; data/restraints/parameters 4019/4/218; GoF = 1.006. Largest peak and hole 3.031 (close to iridium atoms) and -2.788 e/ Å^3.

Crystal data for **9**: $C_{36}H_{41}N_5ORh_2$, M_W 765.56, orange, irregular block (0.203 x 0.155 x 0.152 mm^3), triclinic, space group P-1, *a*: 10.8575(9) Å, *b*: 12.0580(10) Å, *c*: 12.8580(10) Å, *α*: 111.1450(10)°, *β*: 92.3650(10)°, *γ*: 90.4460(10)°, *V* = 1568.2(2) Å^3, *Z* = 2, *Z'* = 1, D_{calc}: 1.621 g cm^{-3}, F(000): 780, T = 100(2) K, μ 1.090 mm^{-1}. 16896 measured reflections (2θ: 3-57°, ω scans 0.3°), 8162 unique (R_{int} = 0.0176); min./max. transm. Factors 0.752/0.862. Final agreement factors were R^1 = 0.0228 (7335 observed reflections, I > 2σ(I)) and wR^2 = 0.0581; data/restraints/parameters 8162/0/426; GoF = 1.019. Largest peak and hole 0.691 (close to rhodium atoms) and -0.348 e/ Å^3.

Crystal data for **11**: $C_{35}H_{40}N_4O_2Rh_2$, 0.25(C_5H_{12}), M_W 772.56, red, irregular block (0.168 x 0.150 x 0.141 mm^3), trigonal, space group R-3, *a*: 30.3580(14) Å, *b*: 30.3580(14) Å, *c*: 18.588(2) Å, *V* = 14836(2) Å^3, *Z* = 18, *Z'* = 1, D_{calc}: 1.557 g cm^{-3}, F(000): 7101, T = 100(2) K, μ 1.039 mm^{-1}. 39077 measured reflections (2θ: 3-57°, ω scans 0.3°), 7950 unique (R_{int} = 0.0938); min./max. transm. Factors 0.739/0.862. Final agreement factors were R^1 = 0.0402 (4270 observed reflections, I > 2σ(I)) and wR^2 = 0.0939; data/restraints/parameters 7950/7/435; GoF = 0.812. Largest peak and hole 0.831 (close to rhodium atoms) and -0.630 e/ Å^3.

Crystal data for **12**: $C_{44}H_{54}Ir_3N_5O$ x 1.5(C_6H_6), M_W 1362.68, colourless, irregular block (0.176 x 0.070 x 0.045 mm^3), monoclinic, space group $P2_1/n$, *a*: 12.1775(12) Å, *b*: 15.1208(15) Å, *c*: 24.793(2) Å, *β*: 96.341(2)°, *V* = 4537.3(8) Å^3, *Z* = 4, *Z'* = 1, D_{calc}: 1.995 g cm^{-3}, F(000): 2620, T = 100(2) K, μ 8.823 mm^{-1}. 71804 measured reflections (2θ: 3-

57°, ω scans 0.3°), 12353 unique (R_{int} = 0.0549); min./max. transm. Factors 0.636/0.862. Final agreement factors were R^1 = 0.0283 (9694 observed reflections, I > 2σ(I)) and wR^2 = 0.0622; data/restraints/parameters 12353/0/ 597; GoF = 1.018. Largest peak and hole 2.021 (close to iridium atoms) and -1.059 e/ Å^3.

Electrochemical Data:

Cyclic voltammograms were recorded in a Metrohm Autolab Potentiostat model PGSTAT302N using a glassy carbon working electrode, platinum wire counter electrode and Ag/AgCl (KCl 3M) reference electrode. Samples were prepared in dry and degassed dichloromethane in a 10^{-3} M analyte concentration containing $[N(^nBu)_4]PF_6$ as supporting electrolyte (10^{-1} M). Measurements were recorded under Ar at a scan rate of 100 mV/s. Potentials are referenced vs Ag/AgCl.

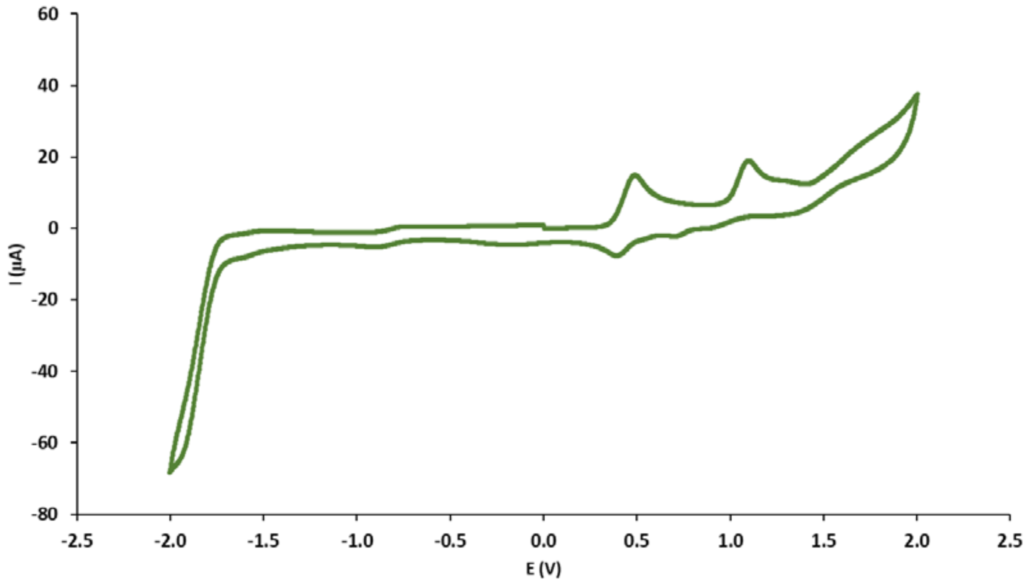

Figure S1. Cyclic voltammogram (initial sweep to positive potentials) of complex **11** (10^{-3} M in CH_2Cl_2, 10^{-1} M $[N(^nBu)_4]PF_6$, counter-electrode: Pt wire; working electrode: Glassy Carbon; Reference electrode: Ag/AgCl; scan rate: 100 mV/s).

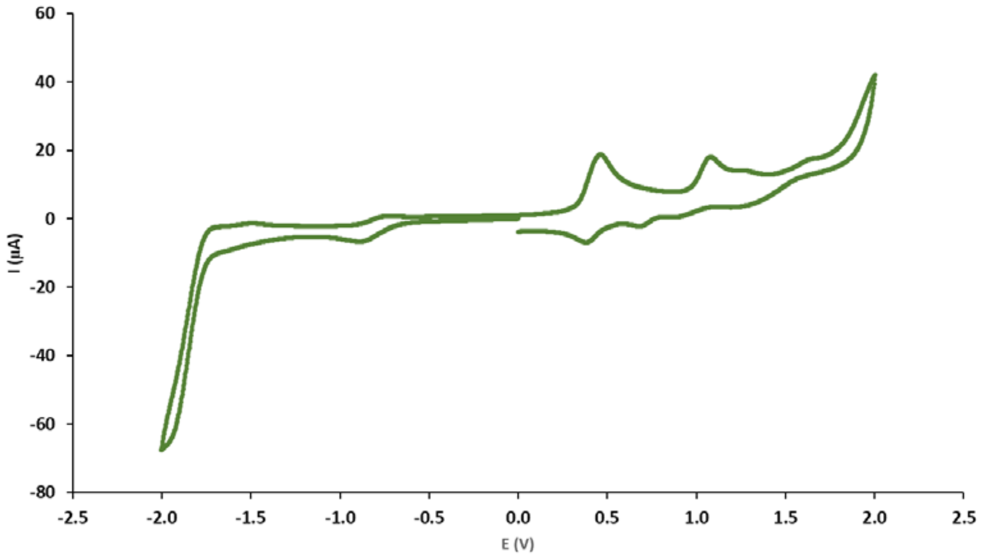

Figure S2. Cyclic voltammogram (initial sweep to negative potentials) of **11** (10^{-3} M in CH_2Cl_2, 10^{-1} M [N(^{n}Bu)$_4$]PF_6, counter-electrode: Pt wire; working electrode: Glassy Carbon; Reference electrode: Ag/AgCl; scan rate: 100 mV/s).

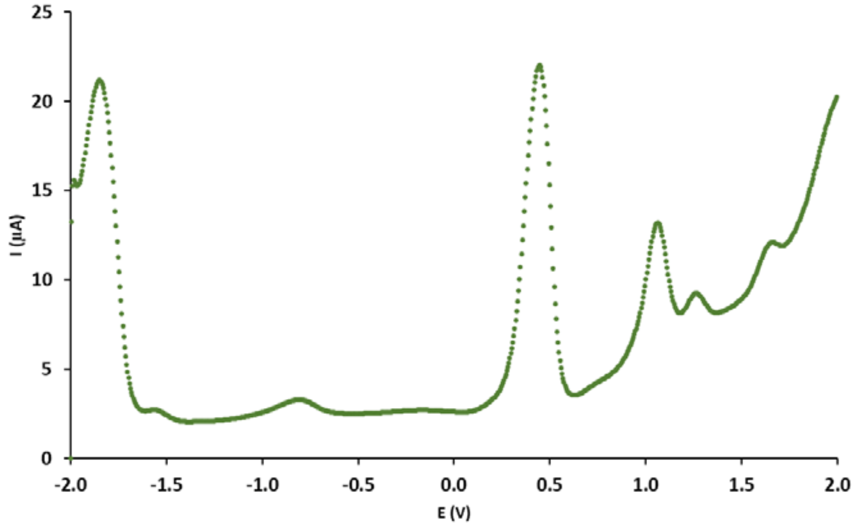

Figure S3. OSWV of compound **11,** 10^{-3} M in CH_2Cl_2, 10^{-1} M [N(^{n}Bu)$_4$]PF_6, counter-electrode: Pt wire; working electrode: Glassy Carbon; Reference electrode: Ag/AgCl; scan rate: 100 mV/s from –2.0 V to 2.0 V.

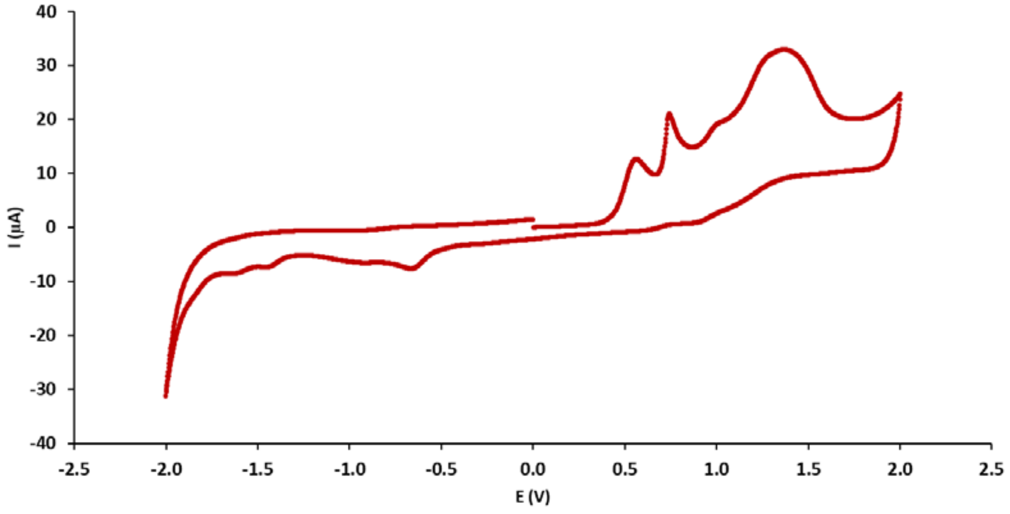

Figure S4. Cyclic voltammogram (initial sweep to positive potentials) of complex **12** (10^{-3} M in CH_2Cl_2, 10^{-1} M $[N(^nBu)_4]PF_6$, counter-electrode: Pt wire; working electrode: Glassy Carbon; Reference electrode: Ag/AgCl; scan rate: 100 mV/s).

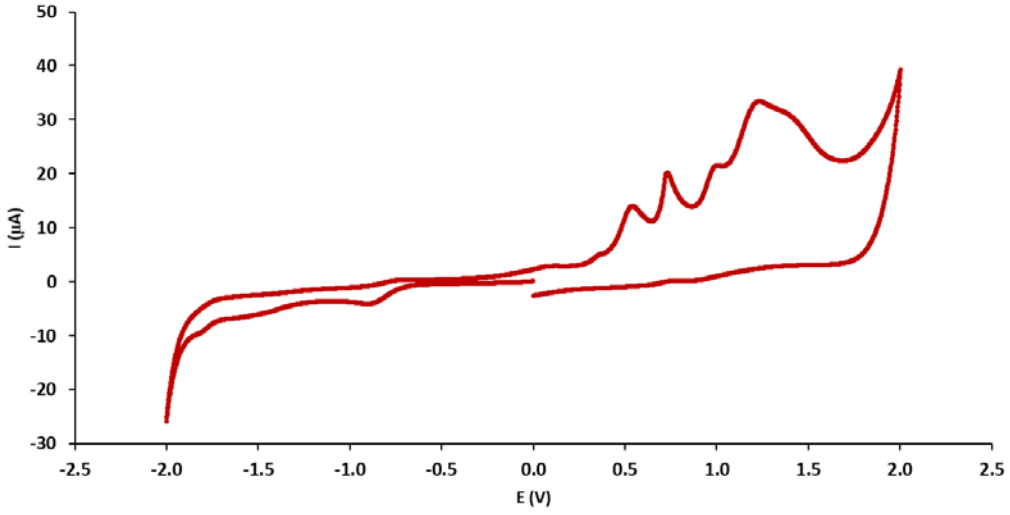

Figure S5. Cyclic voltammogram (initial sweep to negative potentials) of complex **12** (10^{-3} M in CH_2Cl_2, 10^{-1} M $[N(^nBu)_4]PF_6$, counter-electrode: Pt wire; working electrode: Glassy Carbon; Reference electrode: Ag/AgCl; scan rate: 100 mV/s).

Computational Data

Calculations were performed at the DFT level using the M06 functional[3] including an ultrafine integration grid, as implemented in Gaussian 09.[4] The Rh and Ir atoms were described using the scalar relativistic Stuttgart-Dresden SDD pseudopotential and its associated double-ζ basis set,[5] The standard triple-ζ basis set 6-311G(d,p) basis set was used for the rest of atoms.[6] Calculation of the vibrational frequencies[7] at the optimized geometries showed that the compounds are minima on the potential energy surface. This level is denoted M06/6-311G(d,p)&SDD.

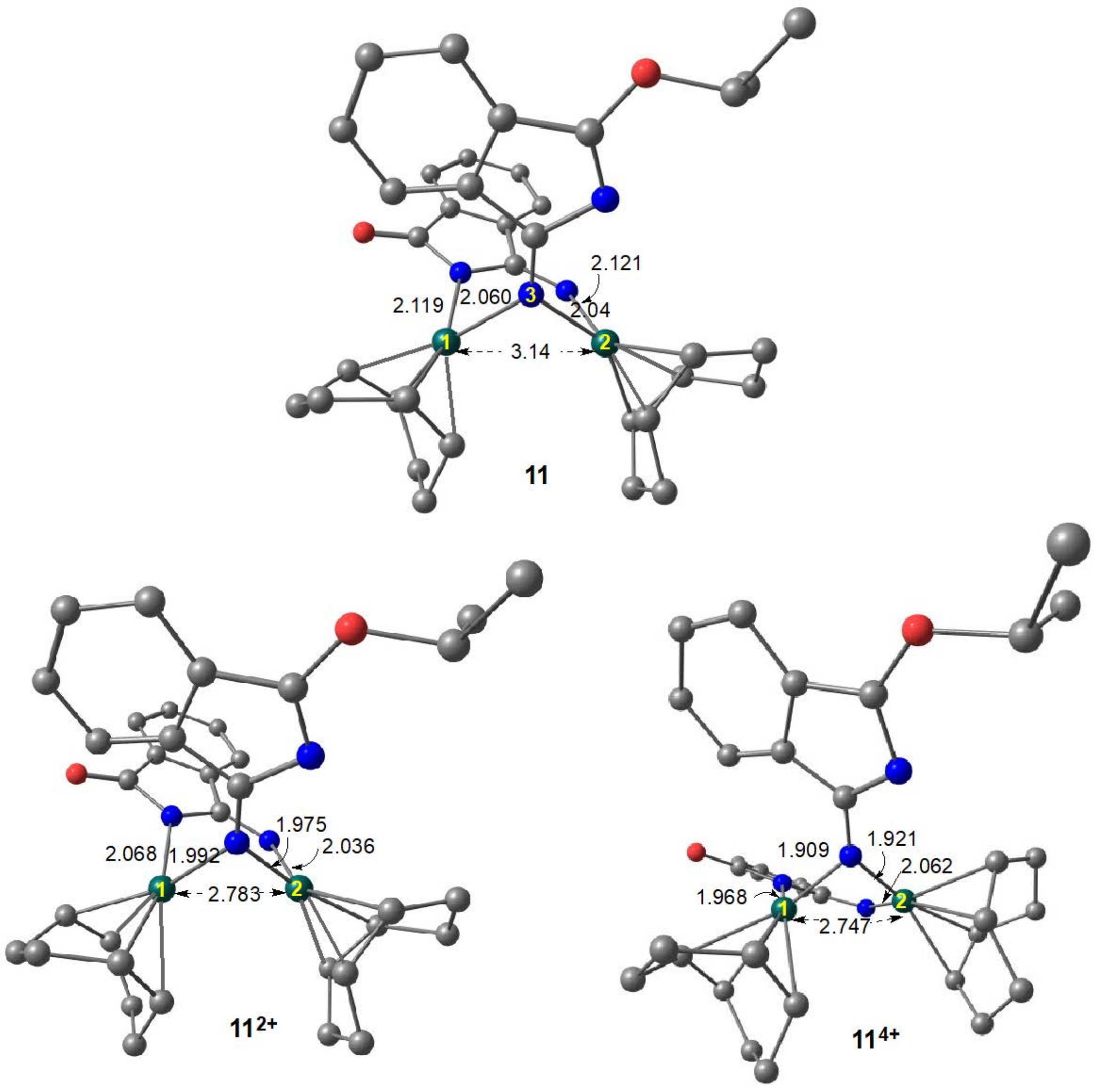

Figure S6. Fully optimized DFT geometries (M06/6-311G(d,p)&SDD(f)) of complex **11** and the corresponding dication $\mathbf{11^{2+}}$ and tetracation $\mathbf{11^{4+}}$. Bond lengths are given in Å. Selected atoms: 1 (Rh(1)); 2 (Rh(2)).

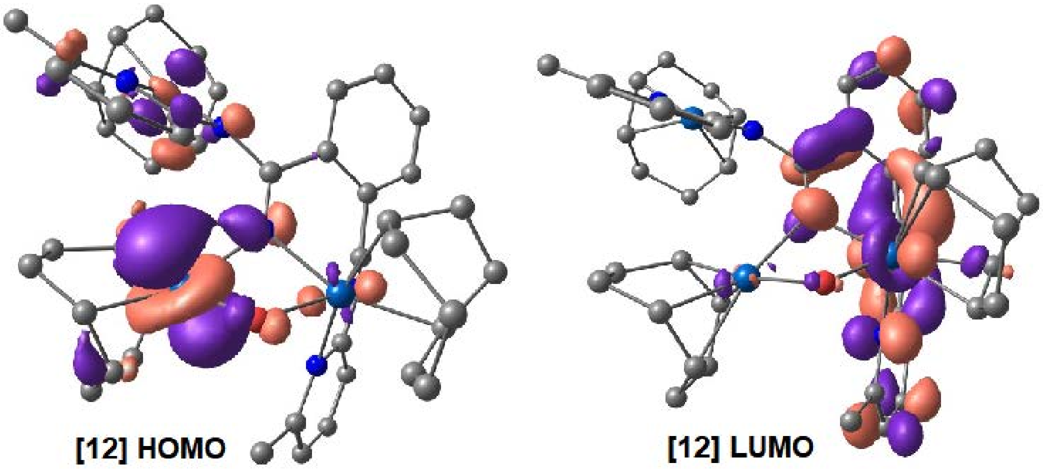

Figure S7. Computed (DFT/M06/6-311G(d,p)&SDD(f)) HOMO and LUMO orbitals for complex **12**. Hydrogen atoms are omitted for clarity. Isosurface value of 0.043.

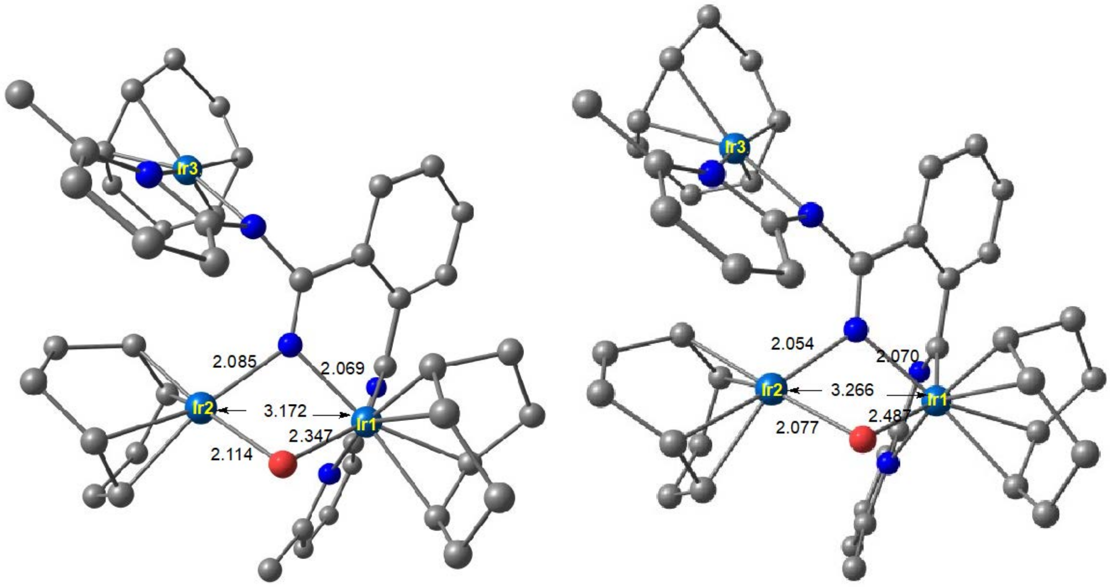

Figure S8. Fully optimized DFT geometries (M06/6-311G(d,p)&SDD(f)) of complex **[12]** (left) and cation radical **[12]**$^{\cdot+}$ (right). H atoms are omitted for clarity. Bond lengths are given in Å.

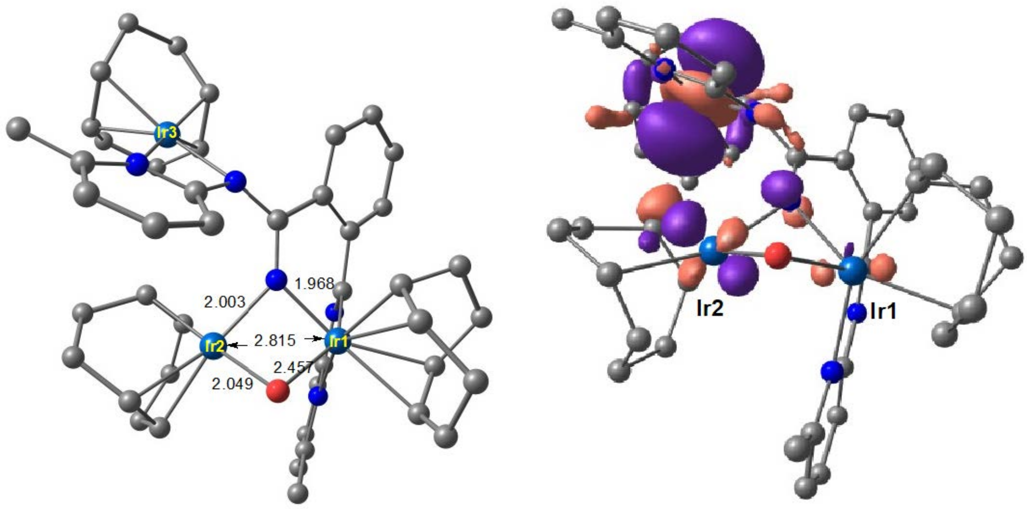

Figure S9. Fully optimized DFT geometry (M06/6-311G(d,p)&SDD(f)) of dication **[12]**$^{2+}$ (left) and the computed HOMO orbital of this species mostly located on the Ir(3) atom (right). H atoms are omitted for clarity. Bond lengths are given in Å. Isosurface value 0.043.

Energies of the optimized complexes

Complex 11

Sum of electronic and zero-point Energies= -1946.978333

Sum of electronic and thermal Energies= -1946.938705

Sum of electronic and thermal Enthalpies= -1946.937761

Sum of electronic and thermal Free Energies= -1947.049809

Dication [11]$^{2+}$

Sum of electronic and zero-point Energies= -1946.408520

Sum of electronic and thermal Energies= -1946.368166

Sum of electronic and thermal Enthalpies= -1946.367222

Sum of electronic and thermal Free Energies= -1946.480533

Tetracation [11]$^{4+}$

Sum of electronic and zero-point Energies= -1944.994009

Sum of electronic and thermal Energies= -1944.952557

Sum of electronic and thermal Enthalpies= -1944.951613

Sum of electronic and thermal Free Energies= -1945.068212

Complex 12

Sum of electronic and zero-point Energies= -2369.488075

Sum of electronic and thermal Energies= -2369.438873

Sum of electronic and thermal Enthalpies= -2369.437929

Sum of electronic and thermal Free Energies= -2369.567626

Radical Cation [12]$^{+}$

Sum of electronic and zero-point Energies= -2368.823838

Sum of electronic and thermal Energies= -2368.775945

Sum of electronic and thermal Enthalpies= -2368.775001

Sum of electronic and thermal Free Energies= -2368.904310

Dication [12]$^{2+}$

Sum of electronic and zero-point Energies= -2368.941145

Sum of electronic and thermal Energies= -2368.891397

Sum of electronic and thermal Enthalpies= -2368.890452

Sum of electronic and thermal Free Energies= -2369.020916

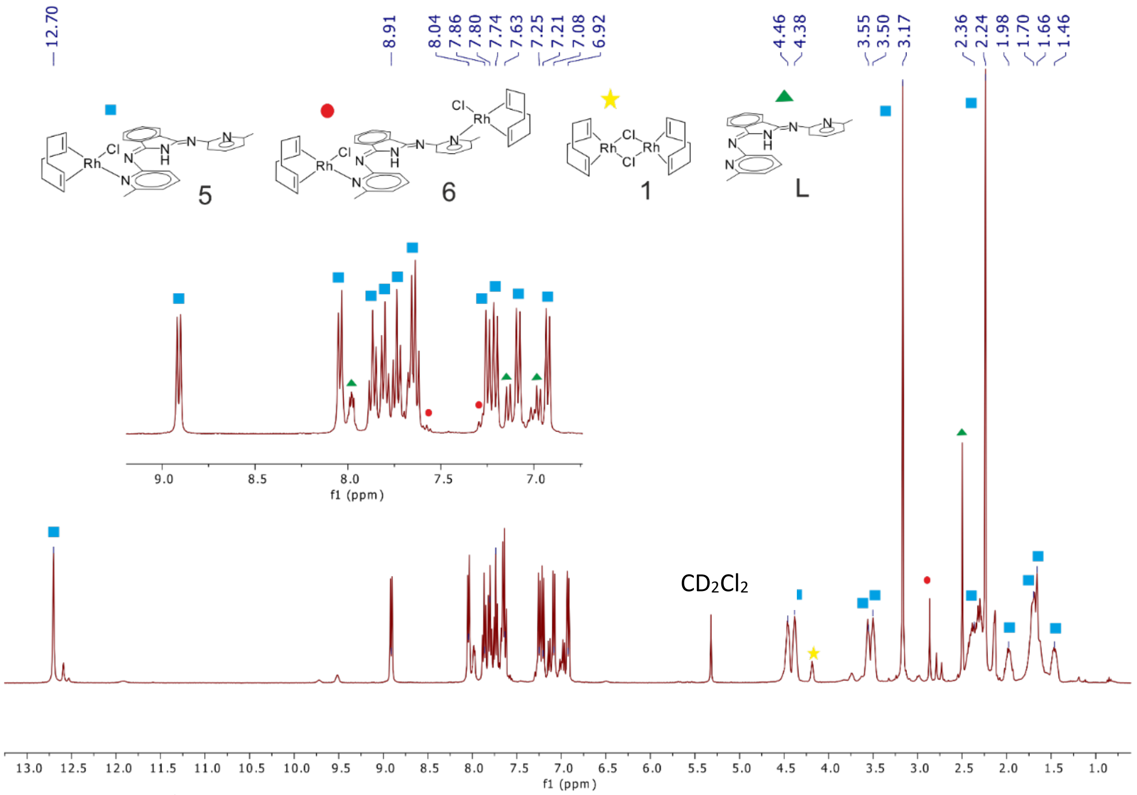

Figure S10. ^{1}H NMR (400 MHz, CD_2Cl_2, 213 K) of the solution resulting of the addition of 2.0 mol of HBMePHI to **1**.

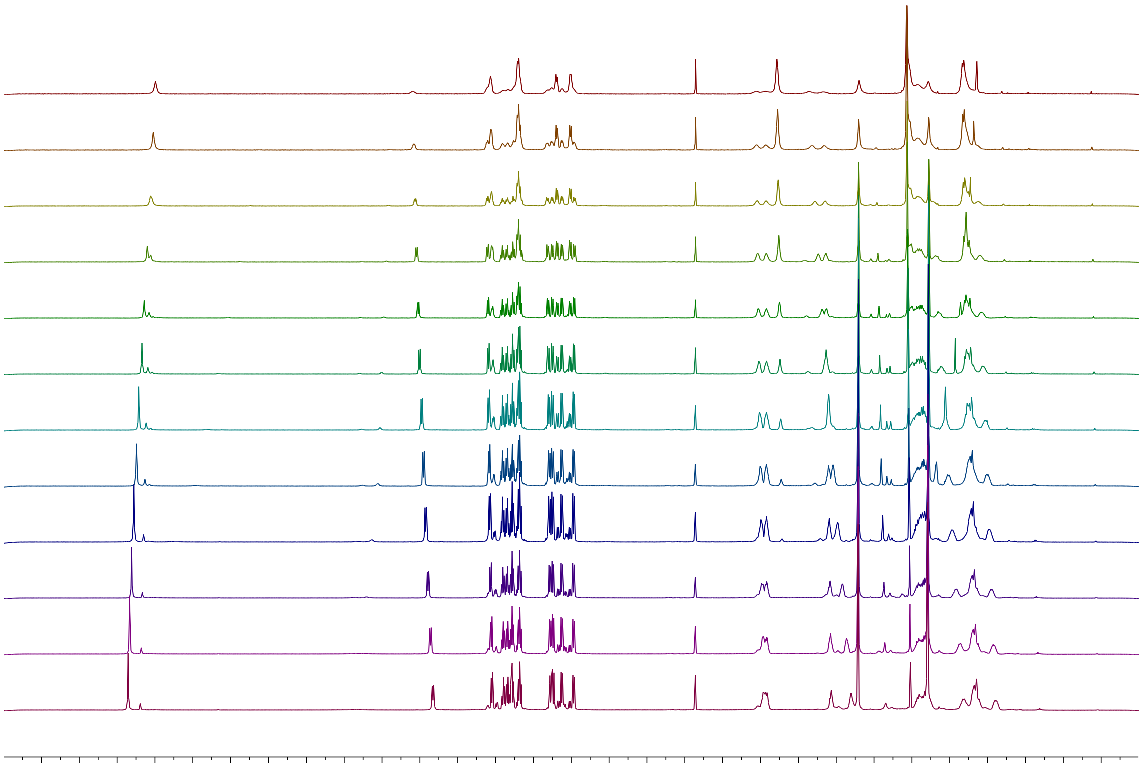

Figure S11. ^{1}H NMR as a function of the temperature (400 MHz, CD_2Cl_2) of the solution resulting of the addition of 2.0 mol of HBMePHI to **1**.

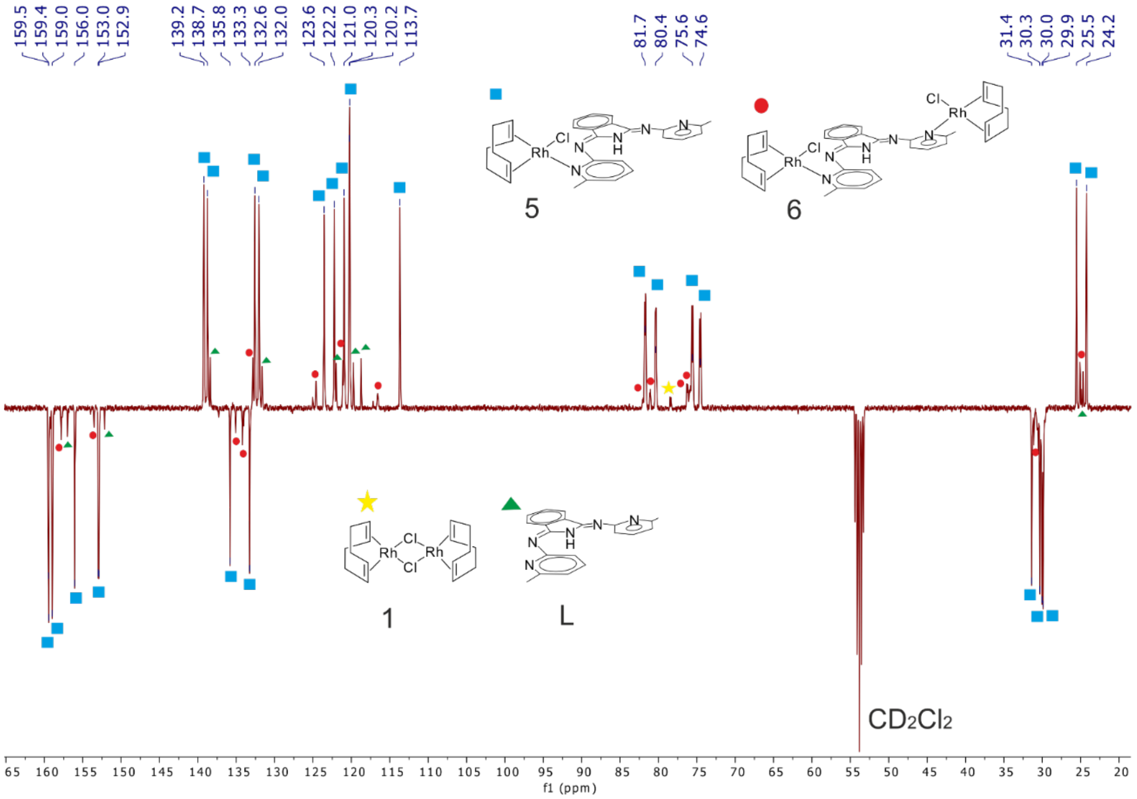

Figure S12. $^{13}C\{^{1}H\}$-APT NMR (100.6 MHz, CD_2Cl_2, 213 K) of the solution resulting of the addition of 2.0 mol of HBMePHI to **1**.

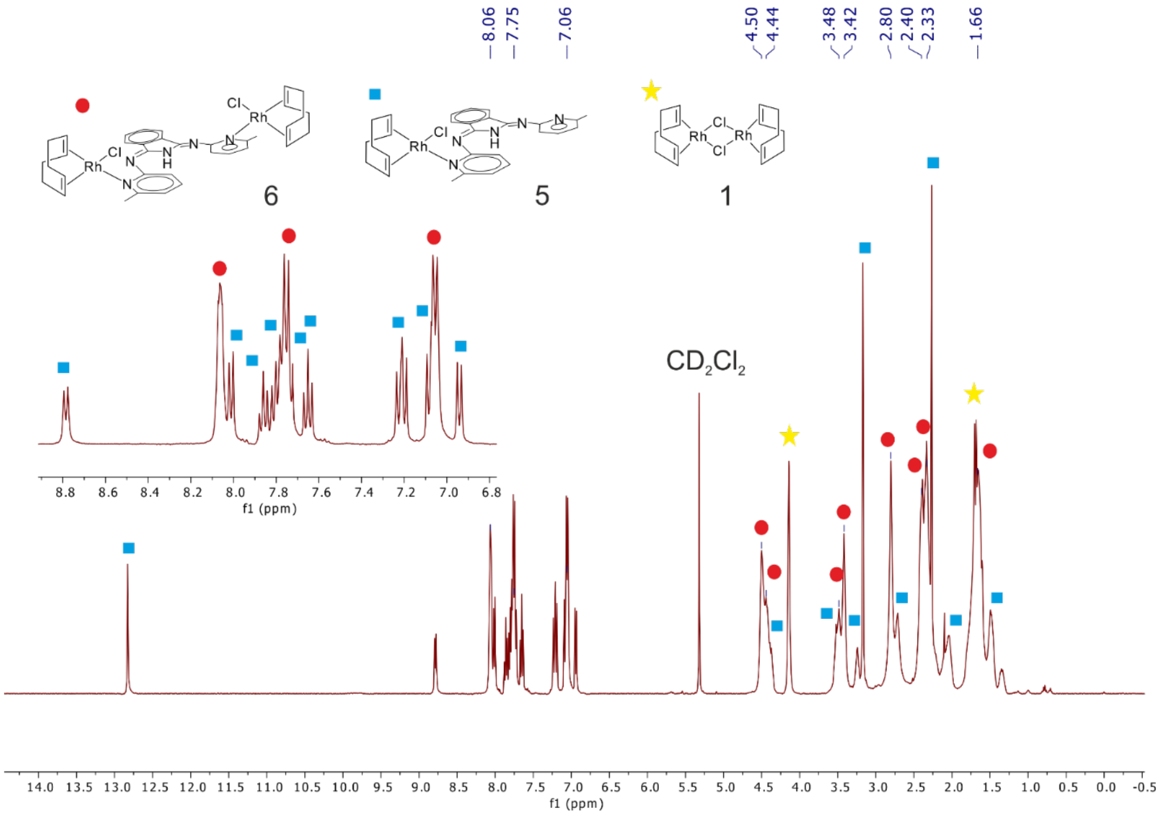

Figure S13. ^{1}H NMR (400 MHz, CD_2Cl_2, 183 K) of the solution resulting of the addition of 1.0 mol of HBMePHI to **1**.

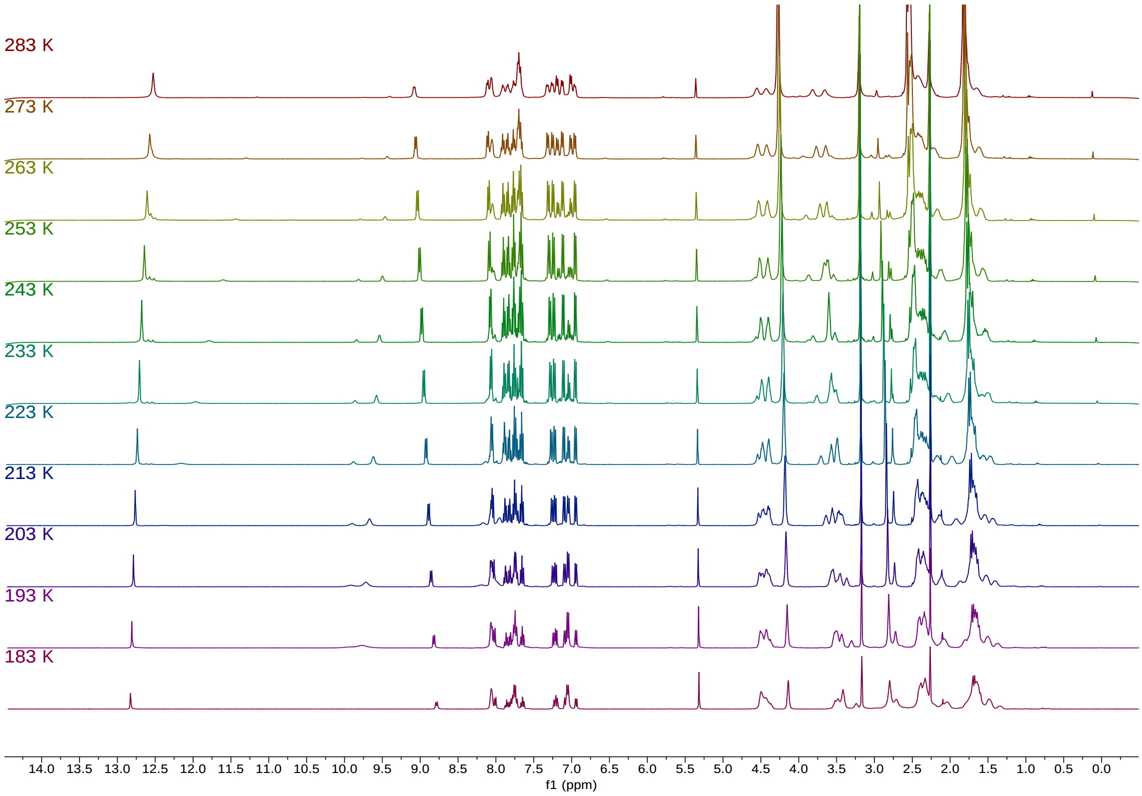

Figure S14. ^{1}H NMR as a function of the temperature (400 MHz, CD_2Cl_2) of the solution resulting of the addition of 1.0 mol of HBMePHI to **1**.

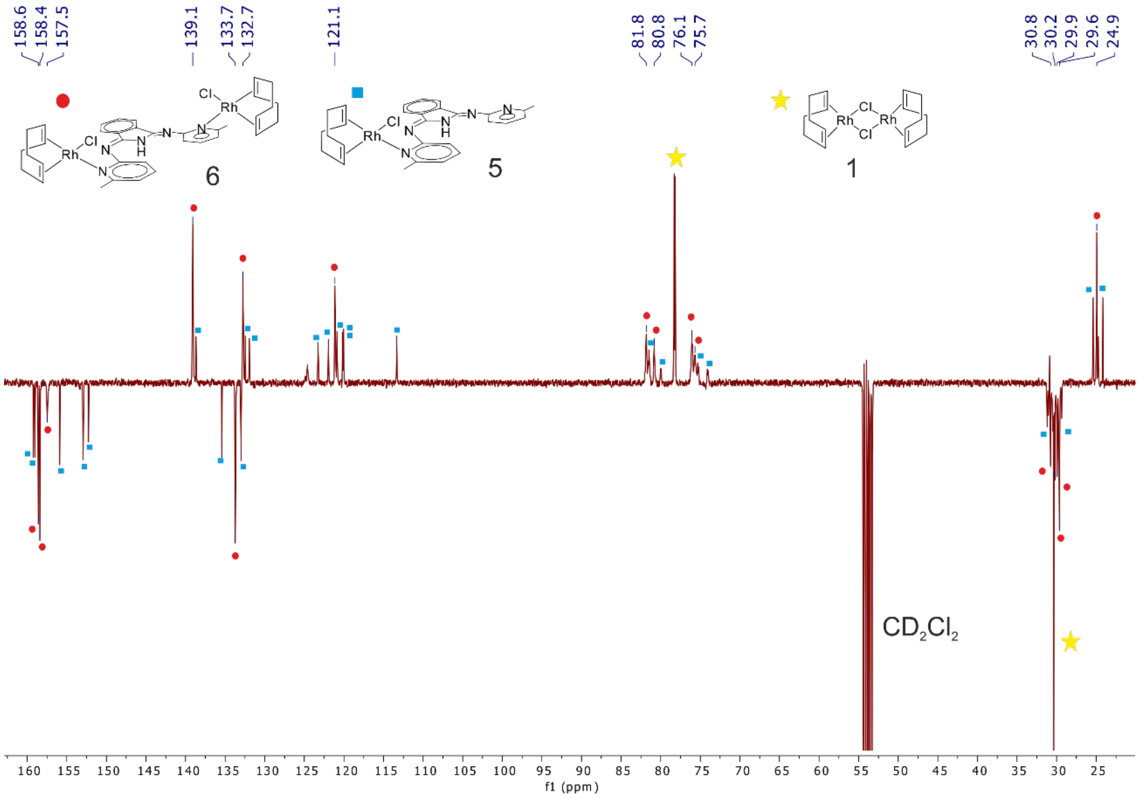

Figure S15. ^{13}C{^{1}H} APT-NMR (100.6 MHz, CD_2Cl_2, 183 K) of the solution resulting of the addition of 1.0 mol of HBMePHI to **1**.

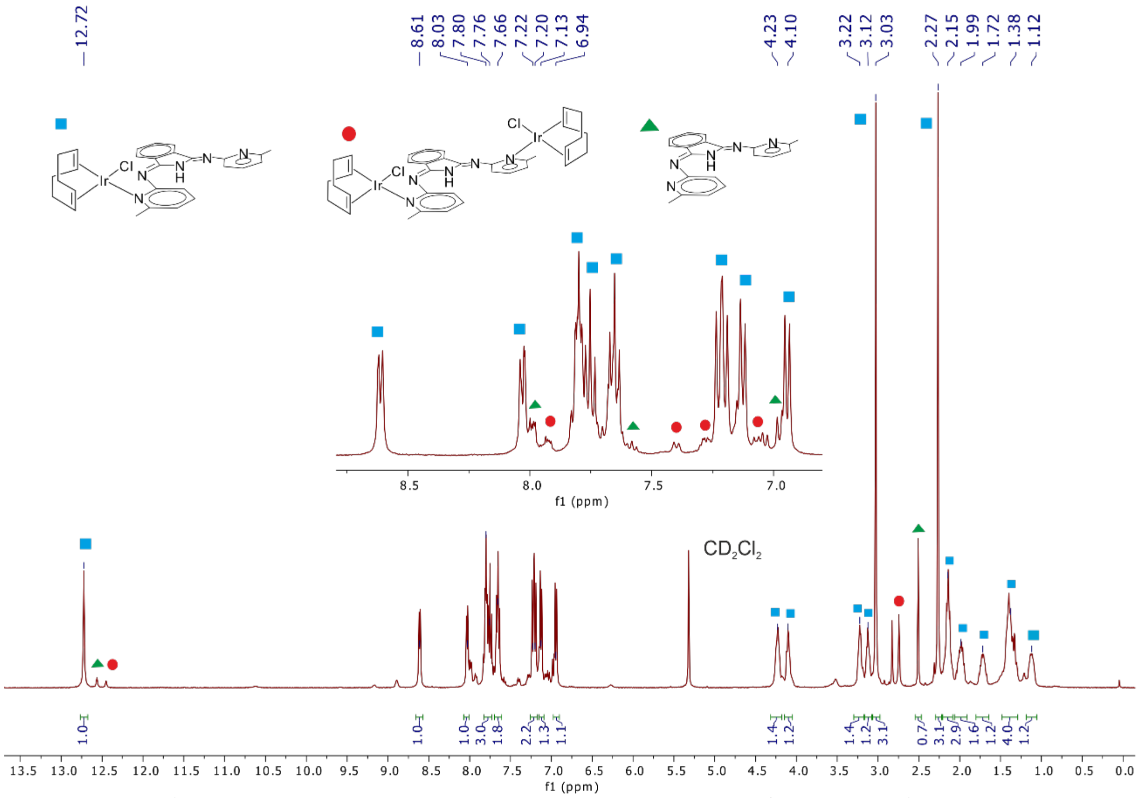

Figure S16. ^{1}H NMR (400 MHz, CD_2Cl_2, 243 K) of IrCl(η^4-C_8H_{12}){κ^1-N_{py}-(HBMePHI)}(**7**).

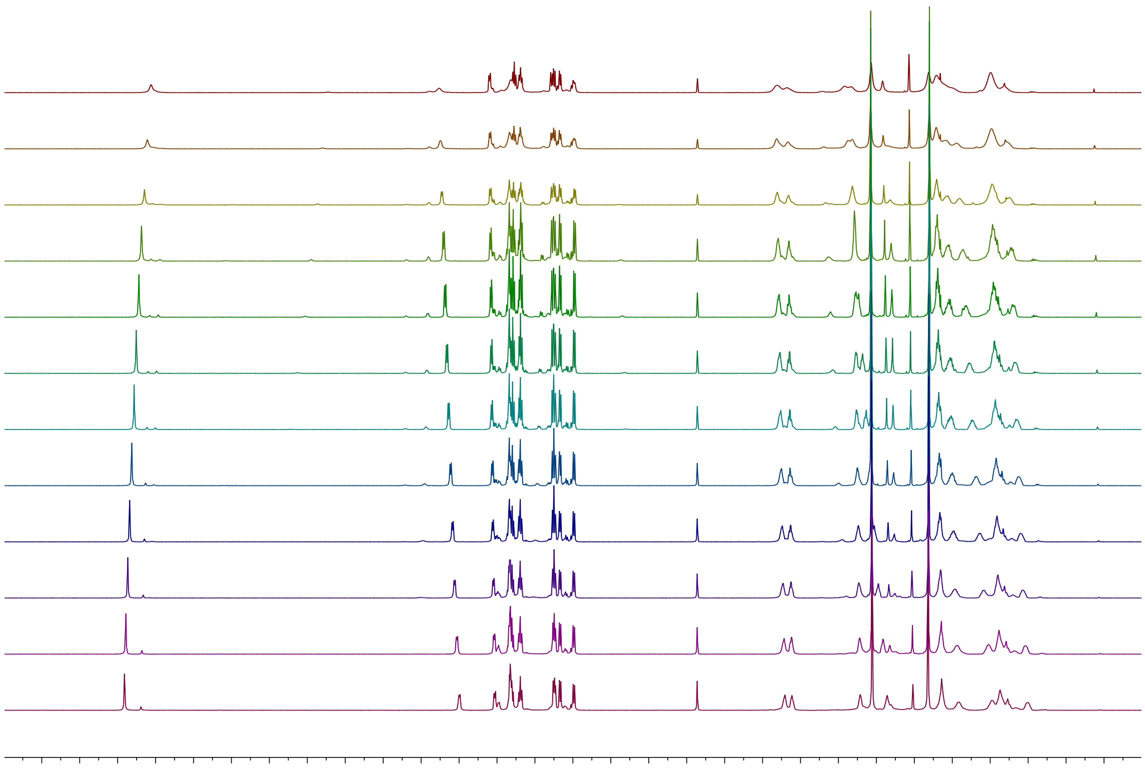

Figure S17. ^{1}H NMR as a function of the temperature (400 MHz, CD_2Cl_2) of IrCl(η^4-C_8H_{12}){κ^1-N_{py} -(HBMePHI)}(**7**).

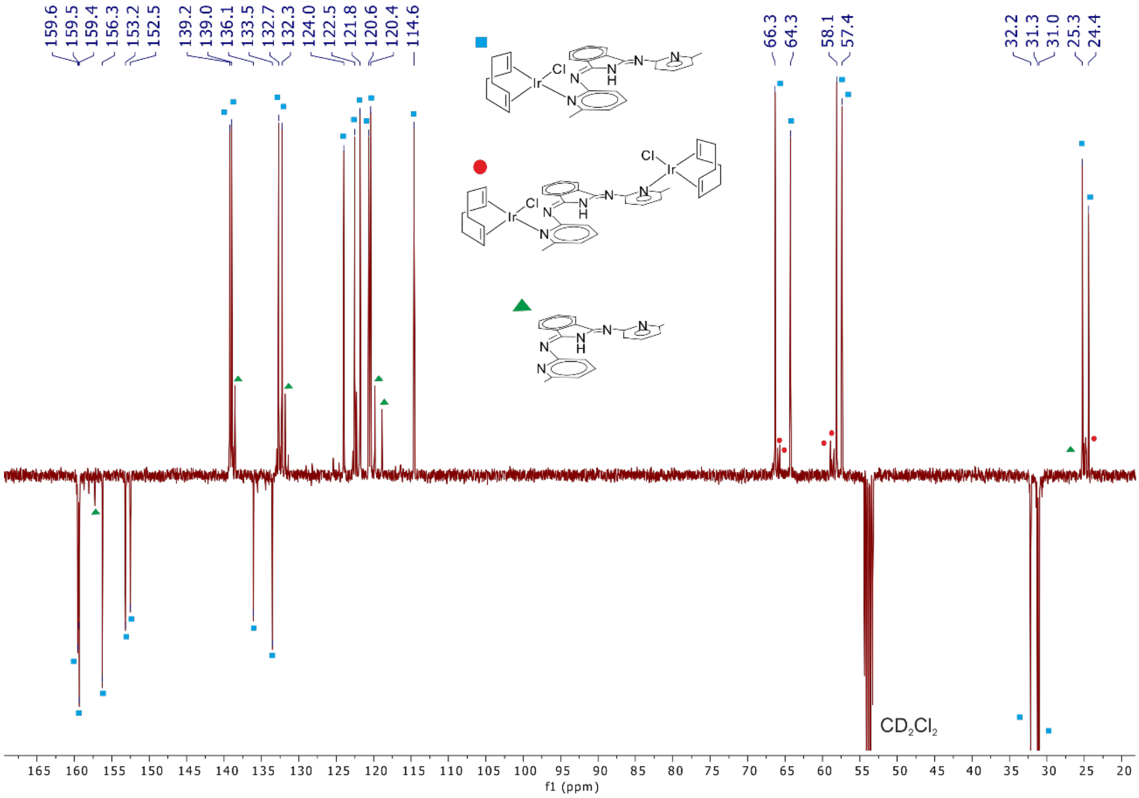

Figure S18. $^{13}C\{^1H\}$-APT NMR (100.6 MHz, CD_2Cl_2, 243 K) of IrCl(η^4-C_8H_{12}){κ^1-N_{py}-(HBMePHI)}(**7**).

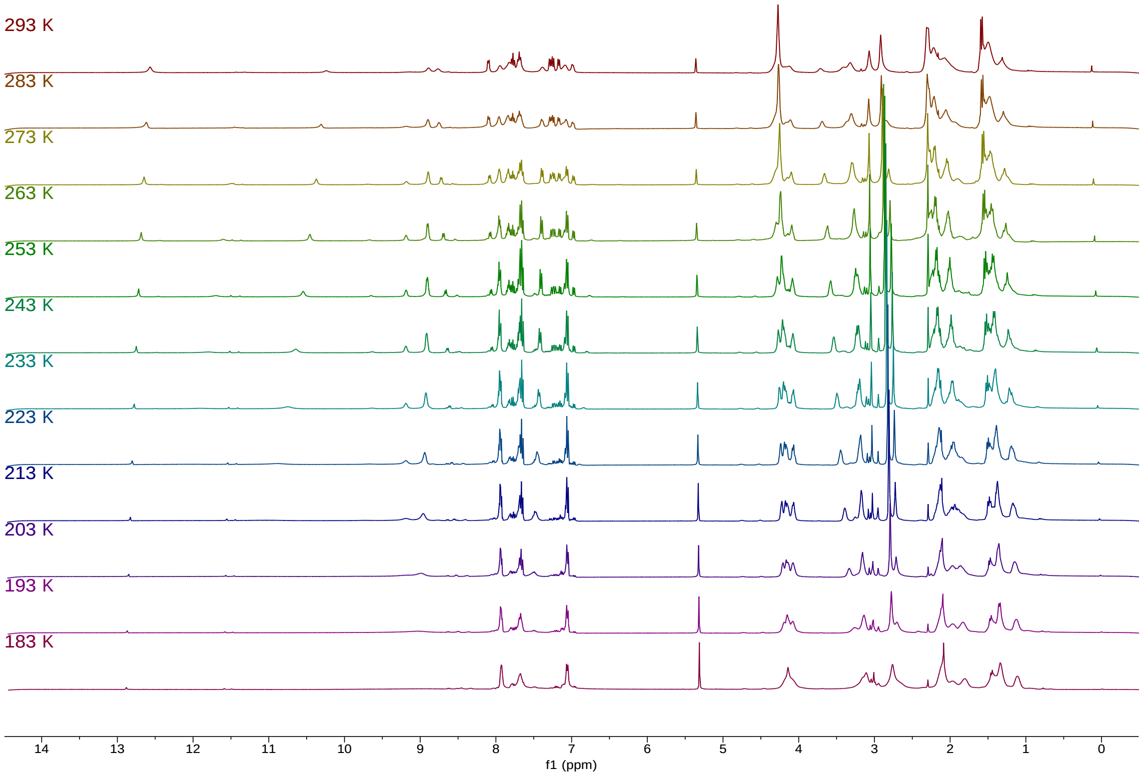

Figure S19. ^1H NMR as a function of the temperature (400 MHz, CD_2Cl_2) of [IrCl(η^4-C_8H_{12})]$_2${μ-N_{py},N_{py}-(HBMePHI)} (**8**).

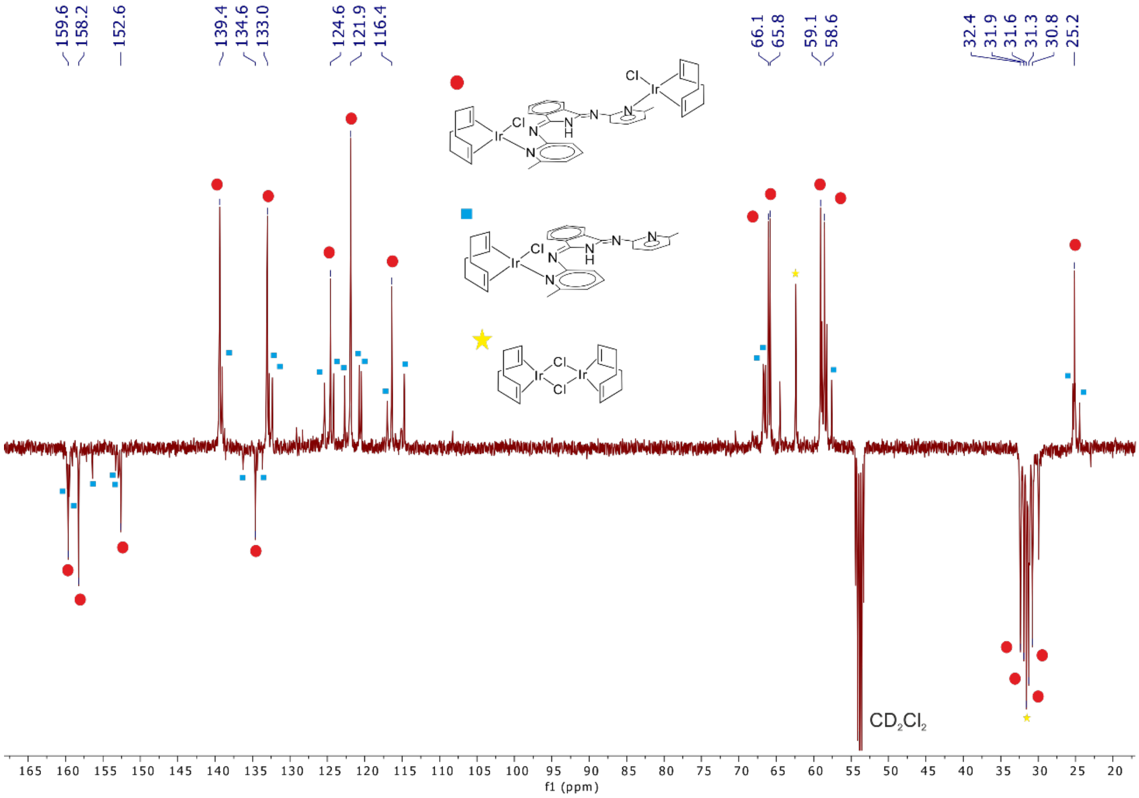

Figure S20. High field region of the $^{13}C\{^1H\}$-APT NMR (100.6 MHz, CD_2Cl_2, 253 K) of $[IrCl(\eta^4\text{-}C_8H_{12})]_2\{\mu\text{-}N_{py},N_{py}\text{-(HBMePHI)}\}$ (**8**).

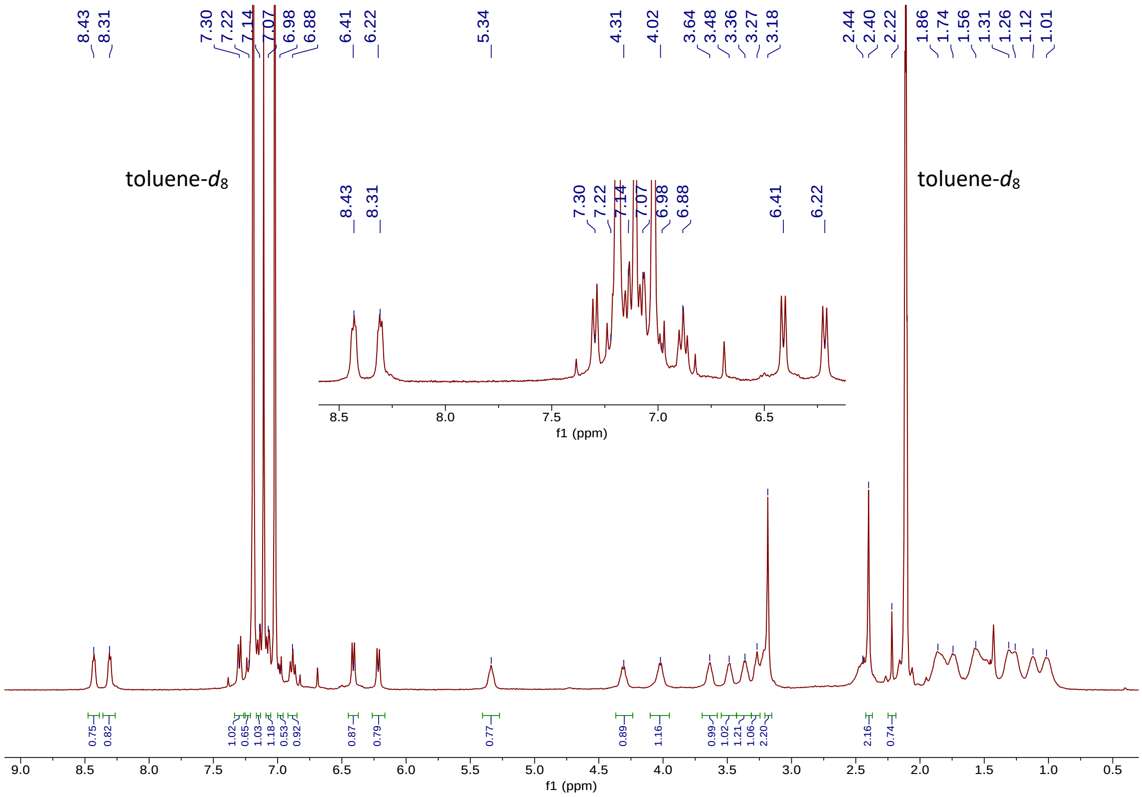

Figure S21. ^1H NMR (400 MHz, toluene-d_8, 183 K) of $[Rh(\eta^4\text{-}C_8H_{12})]_2(\mu\text{-OH})\{\mu\text{-}N_{iso},N_{py}\text{-(BMePHI)}\}$ (**9**).

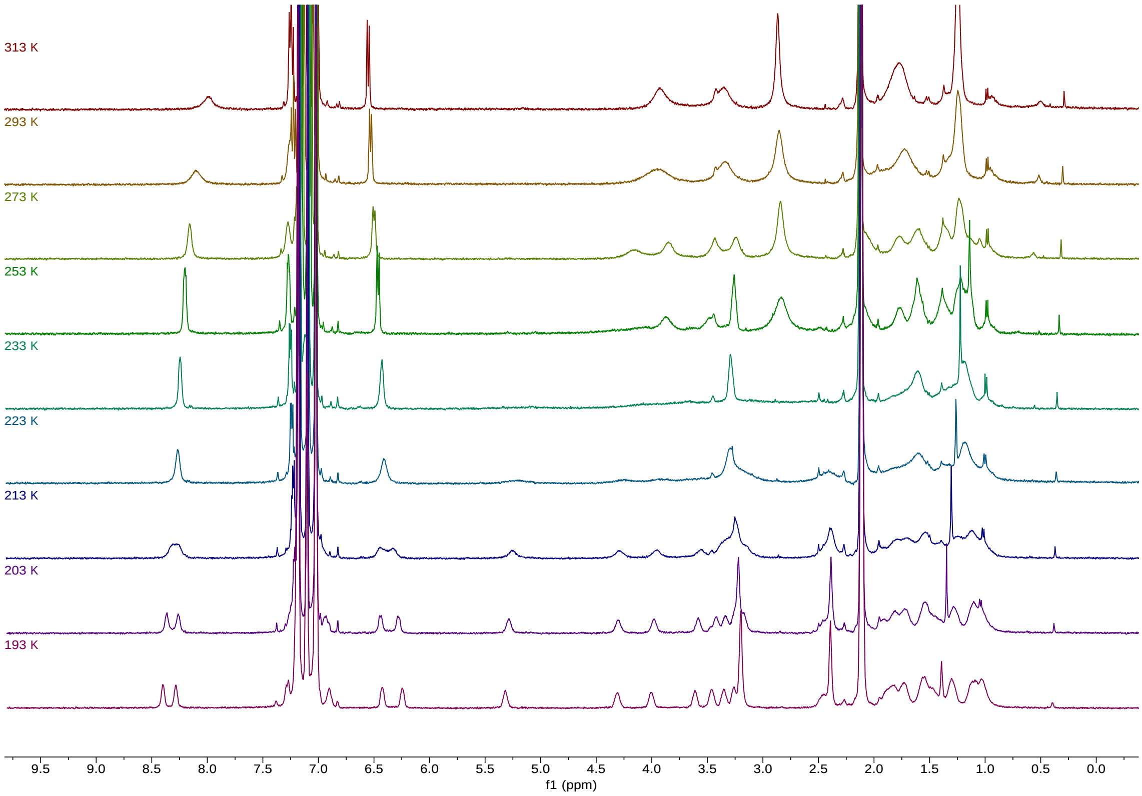

Figure S22. ^{1}H NMR as a function of the temperature (400 MHz, toluene-d_8) of [Rh(η^4-C_8H_{12})]$_2$(μ-OH){μ-N_{iso},N_{py}-(BMePHI)} (**9**).

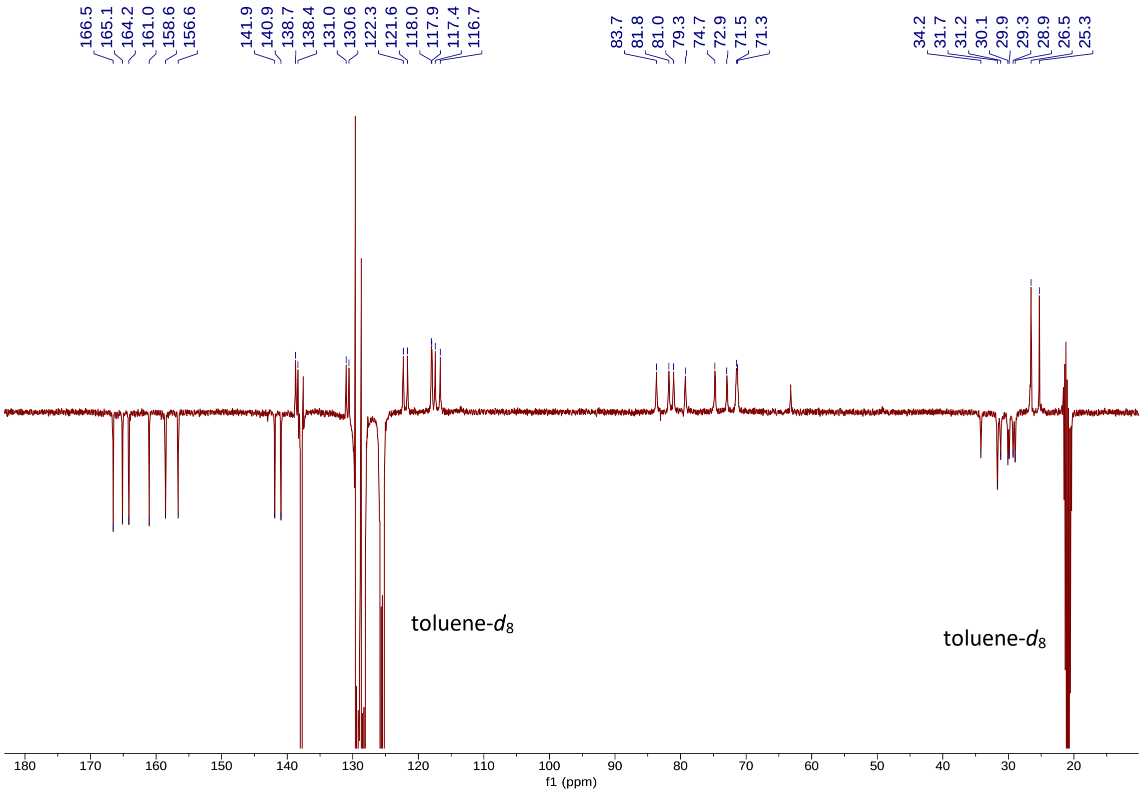

Figure S23. ^{13}C{^{1}H}-APT NMR (100.6 MHz, toluene-d_8, 193 K) of [Rh(η^4-C_8H_{12})]$_2$(μ-OH){μ-N_{iso},N_{py}-(BMePHI)} (**9**).

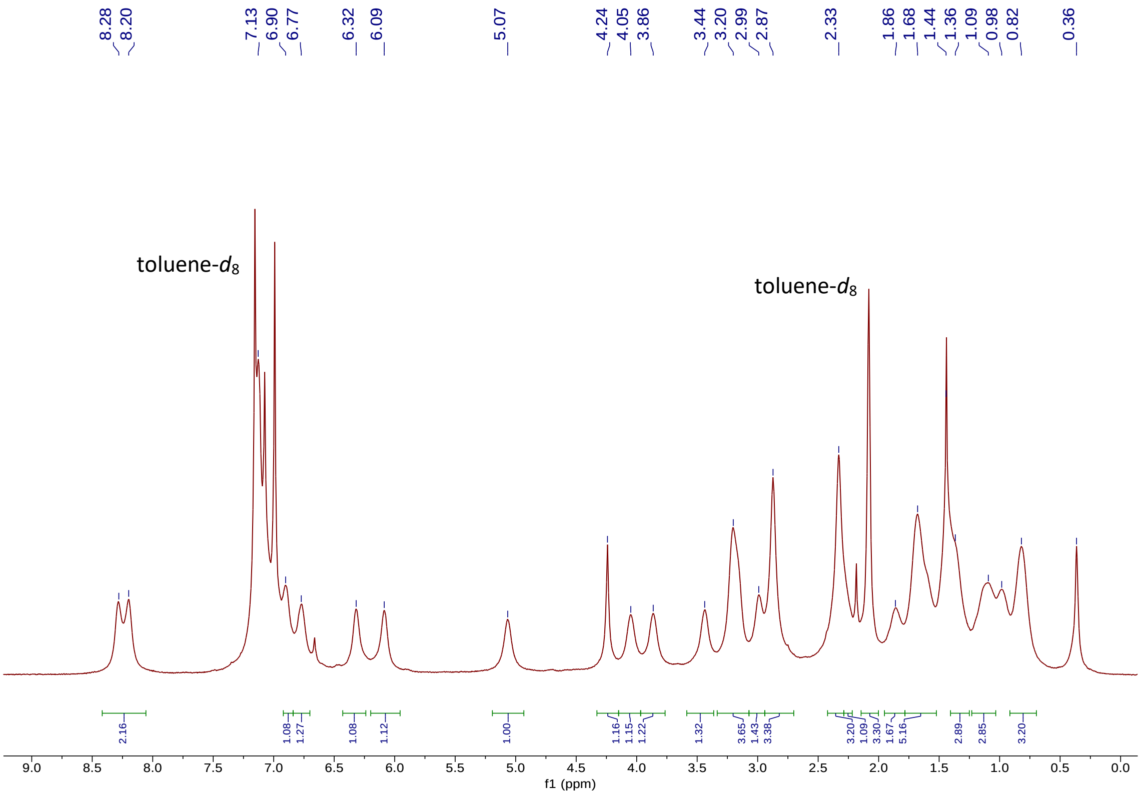

Figure S24. ¹H NMR (400 MHz, toluene-d_8, 193 K) of $[Ir(\eta^4\text{-}C_8H_{12})]_2(\mu\text{-}OH)\{\mu\text{-}N_{iso},N_{py}\text{-}(BMePHI)\}$ (**10**).

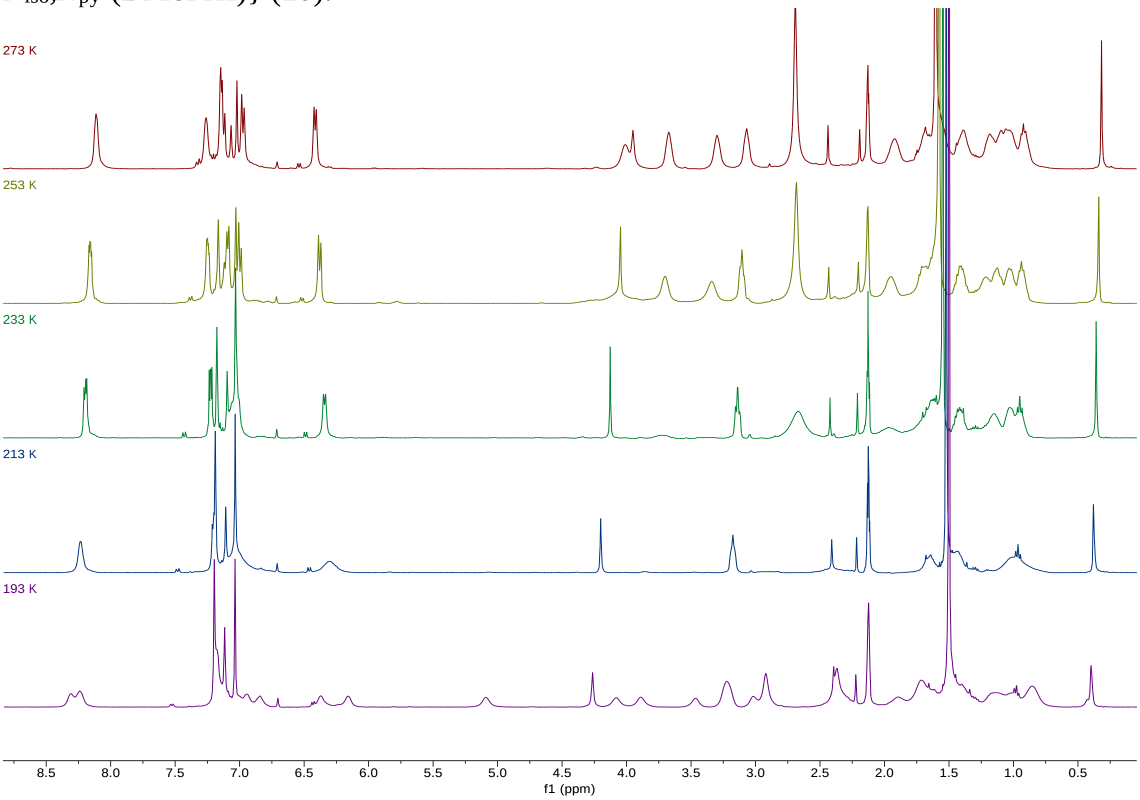

Figure S25. ¹H NMR as a function of the temperature (400 MHz, toluene-d_8) of $[Ir(\eta^4\text{-}C_8H_{12})]_2(\mu\text{-}OH)\{\mu\text{-}N_{iso},N_{py}\text{-}(BMePHI)\}$ (**10**).

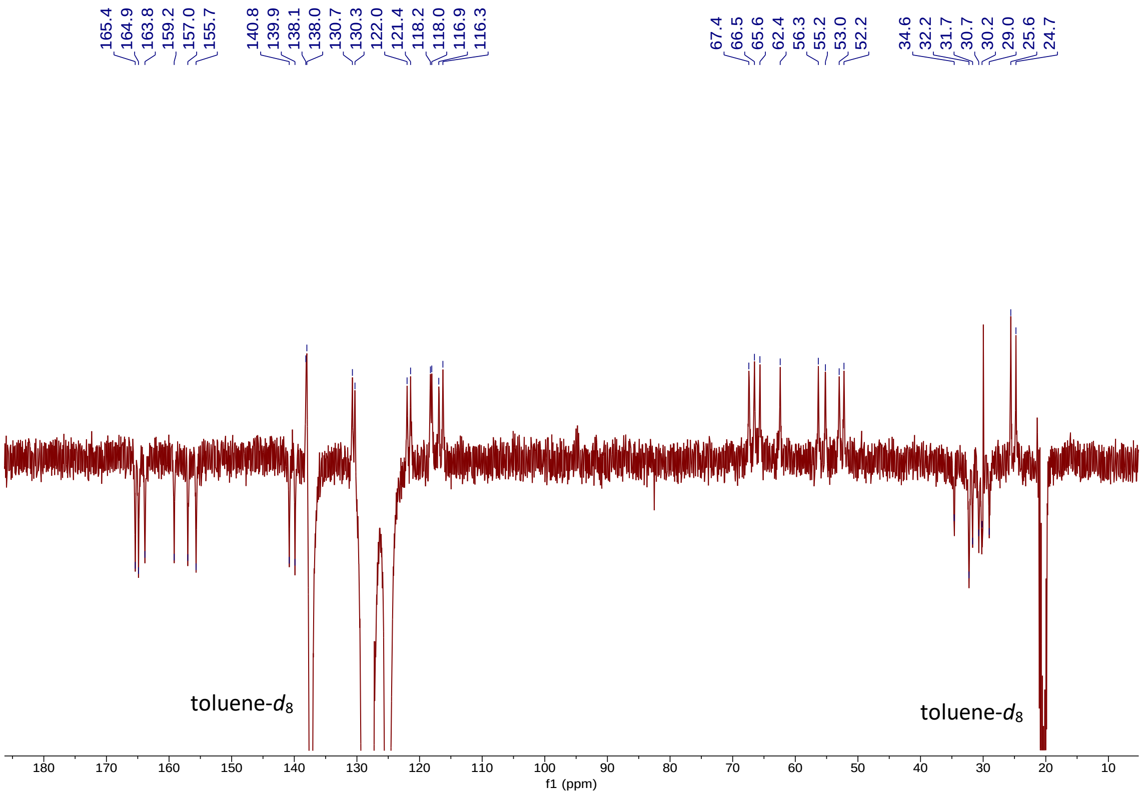

Figure S26. $^{13}C\{^1H\}$-APT NMR (100.6 MHz, toluene-d_8, 188 K) of [Ir(η^4-C_8H_{12})]$_2$(μ-OH){μ-N_{iso},N_{py}-(BMePHI)} (**10**).

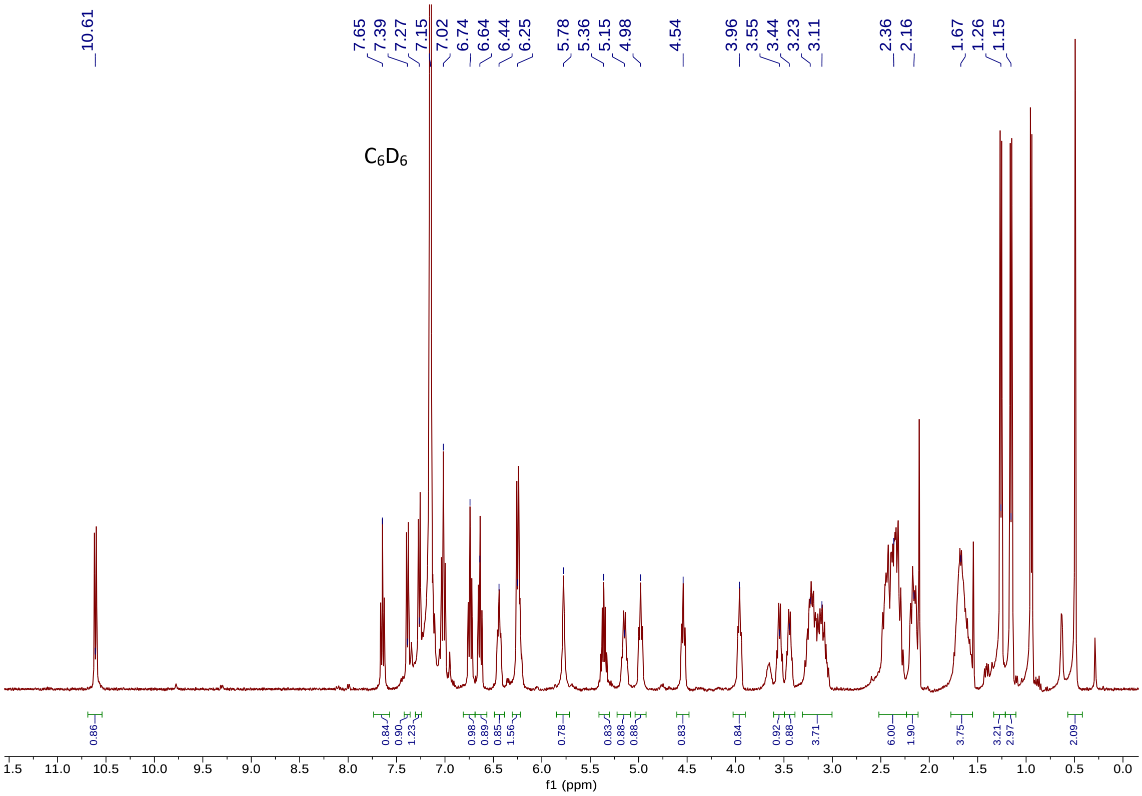

Figure S27. ^1H NMR (400 MHz, C_6D_6, 298 K) of [Rh(η^4-C_8H_{12})]$_2${μ-κ^2-N_{iso},N_{imine}-(HN=C_8H_4NO)}(μ-N=C_8H_4NO^{i}Pr) (**11**).

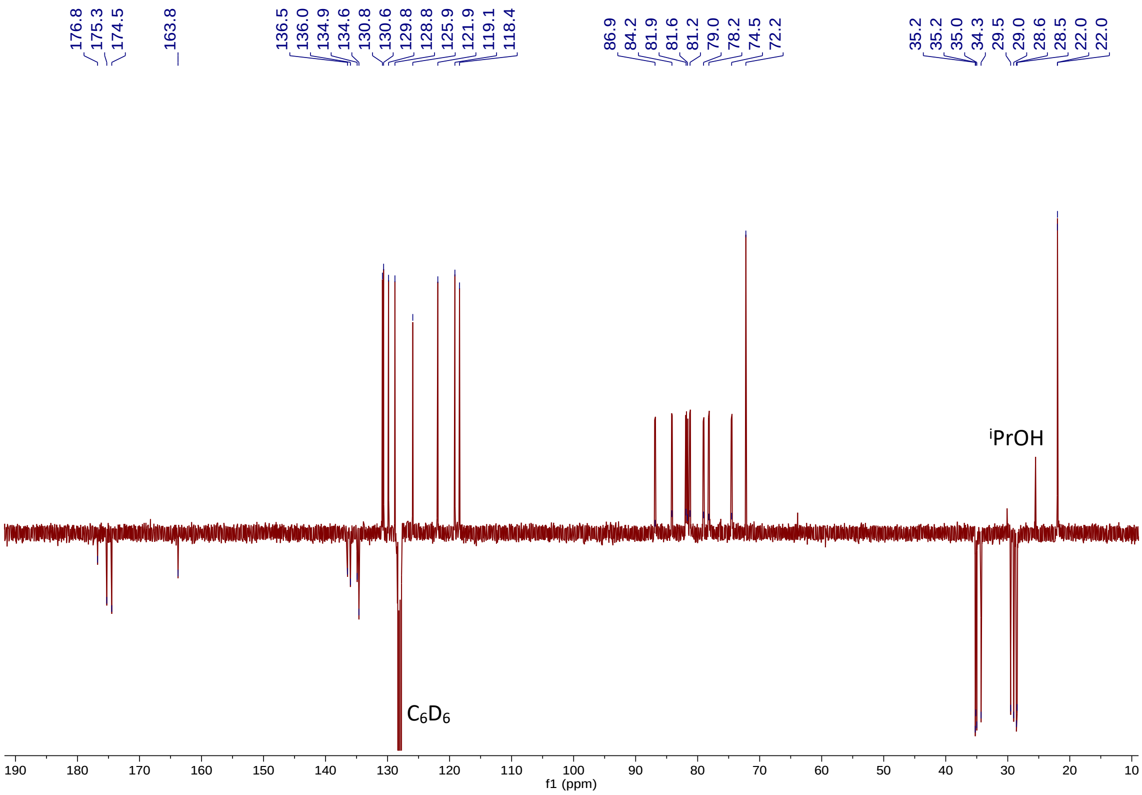

Figure S28. $^{13}C\{^{1}H\}$-APT NMR (100.6 MHz, C_6D_6, 298 K) of $[Rh(\eta^4\text{-}C_8H_{12})]_2\{\mu\text{-}\kappa^2\text{-}N_{iso},N_{imine}\text{-}(HN{=}C_8H_4NO)\}(\mu\text{-}N{=}C_8H_4NO^{i}Pr)$ (**11**).

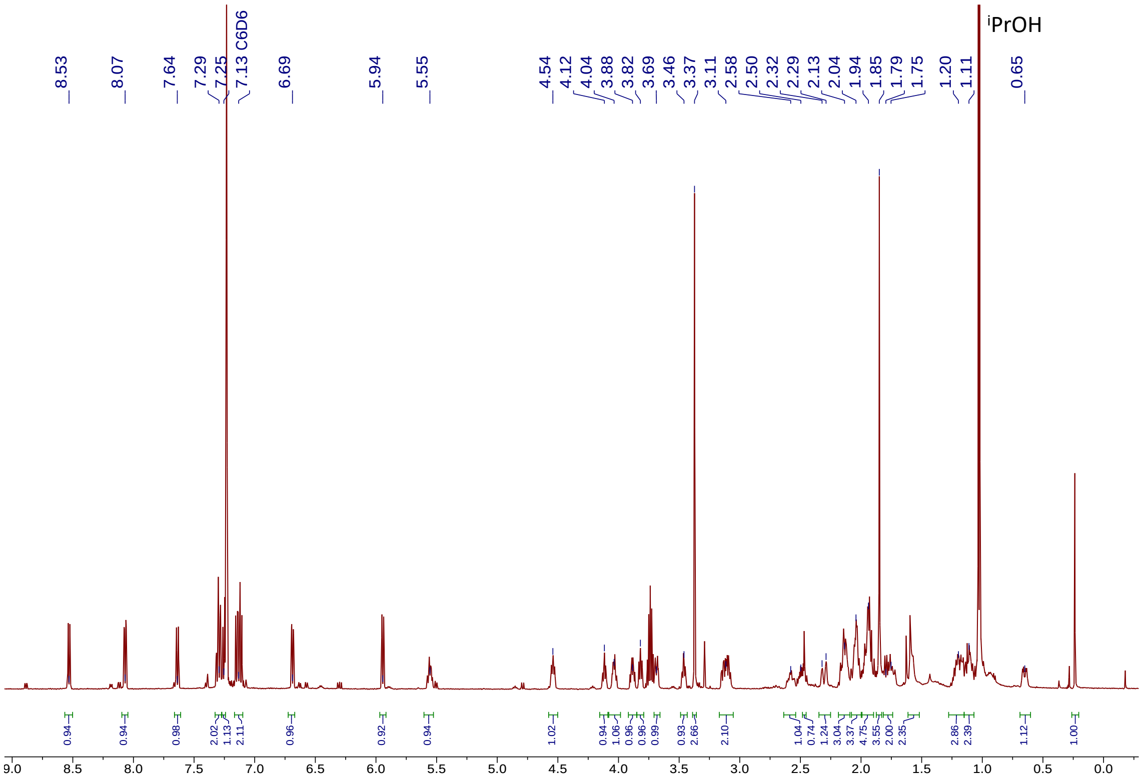

Figure S29. ^{1}H NMR (500.13 MHz, C_6D_6, 298 K) of $Ir_3(\eta^4\text{-}C_8H_{12})_2(\kappa^1\text{-}C,\ \eta^2\text{-}C_8H_{13})(\mu\text{-}OH)(L)$ (**12**).

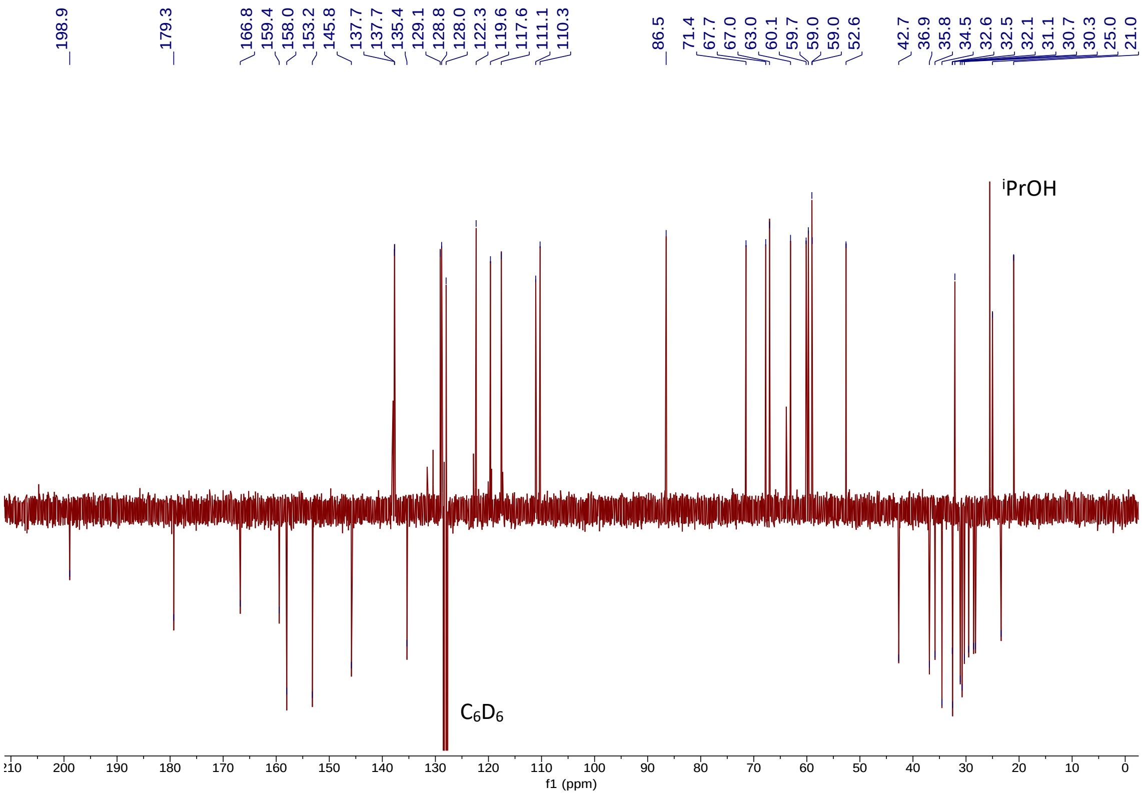

Figure S30. $^{13}C\{^1H\}$-APT NMR (75.5 MHz, C_6D_6, 298 K) of $Ir_3(\eta^4\text{-}C_8H_{12})_2(\kappa^1\text{-}C, \eta^2\text{-}C_8H_{13})(\mu\text{-}OH)(L)$ (**12**).

References

(1) Blessing, R. H. *Acta Crystallogr.* **1995**, *A51*, 33. SADABS: Area-detector absorption correction; Bruker- AXS, Madison, WI, 1996.

(2) SHELXL-2016/6. Sheldrick, G. M. *Acta Cryst.* **2008**, *A64*, 112-122.

(3) Zhao, Y.; Truhlar, D. Theor. *Chem. Acc.* **2008**, *120*, 215−241.

(4) Gaussian 09, Revision B.01; Frisch, M. J.; Trucks, G. W.; Schlegel, H. B.; Scuseria, G. E.; Robb, M. A.; Cheeseman, J. R.; Scalmani, G.; Barone, V.; Mennucci, B.; Petersson, G. A.; Nakatsuji, H.; Caricato, M.; Li, X.; Hratchian, H. P.; Izmaylov, A. F.; Bloino, J.; Zheng, G.; Sonnenberg, J. L.; Hada, M.; Ehara, M.; Toyota, K.; Fukuda, R.; Hasegawa, J.; Ishida, M.; Nakajima, T.; Honda, Y.; Kitao, O.; Nakai, H.; Vreven, T.; J. A., Jr. Montgomery, Peralta, J. E.; Ogliaro, F.; Bearpark, M.; Heyd, J. J.; Brothers, E.; Kudin, K. N.; Staroverov, V. N.; Kobayashi, R.; Normand, J.; Raghavachari, K.; Rendell, A.; Burant, J. C.; Iyengar, S. S.; Tomasi, J.; Cossi, M.; Rega, N.; Millam, N. J.; Klene, M.; Knox, J. E.; Cross, J. B.; Bakken, V.; Adamo, C.; Jaramillo, J.; Gomperts, R.; Stratmann, R. E.; Yazyev, O.; Austin, A. J.; Cammi, R.; Pomelli, C.; Ochterski, J. W.; Martin, R. L.; Morokuma, K.; Zakrzewski, V. G.; Voth, G. A.; Salvador, P.; Dannenberg, J. J.; Dapprich, S.; Daniels, A. D.; Ö. Farkas, Foresman, J. B.; Ortiz, J. V.; Cioslowski, J.; Fox, D. J., Gaussian, Inc.: Wallingford, CT, 2009.

(5) Andrae, D.; Haeussermann, U.; Dolg, M.; Stoll, H.; Preuss, H. *Theor. Chim. Acta* **1990**, *77*, 123−141.

(6) Hehre, W. J.; Ditchfield, R.; Pople, J. A. *J. Chem. Phys.* **1972**, *56*, 2257−2261.

(7) Iver, J. W.; Komornicki, A. K. *J. Am. Chem. Soc.* **1972**, *94*, 2625−2633.
